# A knottin scaffold directs the CXC-chemokine–binding specificity of tick evasins

**DOI:** 10.1074/jbc.RA119.008817

**Published:** 2019-06-05

**Authors:** Angela W. Lee, Maud Deruaz, Christopher Lynch, Graham Davies, Kamayani Singh, Yara Alenazi, James R. O. Eaton, Akane Kawamura, Jeffrey Shaw, Amanda E. I. Proudfoot, João M. Dias, Shoumo Bhattacharya

**Affiliations:** ‡ Radcliffe Department of Medicine Division of Cardiovascular Medicine, University of Oxford, Oxford OX3 7BN, United Kingdom; § Serono Pharmaceutical Research Institute, 1228 Geneva, Switzerland; ¶ Department of Chemistry, University of Oxford, Oxford OX1 3TA, United Kingdom

**Keywords:** chemokine, chemotaxis, crystal structure, inflammation, protein-protein interaction, host-pathogen interaction, evasin, immune response, knottin, tick

## Abstract

Tick evasins (EVAs) bind either CC- or CXC-chemokines by a poorly understood promiscuous or “one-to-many” mechanism to neutralize inflammation. Because EVAs potently inhibit inflammation in many preclinical models, highlighting their potential as biological therapeutics for inflammatory diseases, we sought to further unravel the CXC-chemokine–EVA interactions. Using yeast surface display, we identified and characterized 27 novel CXC-chemokine–binding evasins homologous to EVA3 and defined two functional classes. The first, which included EVA3, exclusively bound ELR^+^ CXC-chemokines, whereas the second class bound both ELR^+^ and ELR^−^ CXC-chemokines, in several cases including C*X*C-motif chemokine ligand 10 (CXCL10) but, surprisingly, not CXCL8. The X-ray crystal structure of EVA3 at a resolution of 1.79 Å revealed a single antiparallel β-sheet with six conserved cysteine residues forming a disulfide-bonded knottin scaffold that creates a contiguous solvent-accessible surface. Swapping analyses identified distinct knottin scaffold segments necessary for different CXC-chemokine–binding activities, implying that differential ligand positioning, at least in part, plays a role in promiscuous binding. Swapping segments also transferred chemokine-binding activity, resulting in a hybrid EVA with dual CXCL10- and CXCL8-binding activities. The solvent-accessible surfaces of the knottin scaffold segments have distinctive shape and charge, which we suggest drives chemokine-binding specificity. These studies provide structural and mechanistic insight into how CXC-chemokine–binding tick EVAs achieve class specificity but also engage in promiscuous binding.

## Introduction

The 45–50 mammalian chemokines are small secreted proteins that are grouped into CC, CXC, XC and CX3C classes based on the spacing between N-terminal cysteine residues. Their chemotactic functions are mediated by binding to a family of G-protein coupled receptors. Chemokines are structurally conserved, with a three-stranded β-sheet, an α-helical segment, and an N-terminal unstructured region (1) along with an N-loop between the second Cys and the β1-strand and 30S and 40S loops between the three β-strands. Certain CXC-chemokines, referred to as “ELR+,” contain a characteristic Glu-Leu-Arg motif in the N-terminal region that binds chemokine receptors CXCR1^7^ and CXCR2 and activates neutrophil migration (2). Binding of chemokines to receptors occurs via chemokine recognition site 1 (CRS1), located in the extracellular N terminus of the receptor, and CRS2, located in the seven-transmembrane bundle. CRS1 binds the proximal N terminus and N-loop/40S loop, whereas CRS2 binds the chemokine distal N terminus (3). The “two-site” model has been refined more recently with the identification of further interaction sites: CRS1.5, between CRS1 and 2, which binds the conserved chemokine disulfide, and CRS0.5, at the receptor distal N terminus, which binds the β1-strand of the chemokine (3). The binding of chemokines to receptors typically involves promiscuous interactions, with several chemokines possessing the ability to bind multiple receptors and, conversely, several receptors having the ability to bind multiple chemokines (4). This phenomenon, together with the expression of a large number of chemokines at sites of inflammation (5) and the expression of several synergistically acting chemokine receptors on inflammatory cells (6, 7), renders the chemokine network robust to attack. It explains, at least in part, the failure of targeting individual chemokines or receptors as a therapeutic strategy for inflammatory disorders (5, 6, 8).

Several parasites and infectious agents overcome the chemokine network and consequent inflammation by producing structurally unrelated proteins that bind, in a promiscuous fashion, to multiple chemokines (8, 9). Viral proteins (9) such as poxvirus CrmD and viral CC-chemokine inhibitor (vCCI), herpesvirus R17 and M3, and papovavirus chemokine-binding protein (CBP), bind multiple chemokines, typically via either the proximal N terminus or via the N-loop/40S loop, preventing binding to CRS1 (3). Ticks draw blood for days to weeks without eliciting inflammation (10), and investigation of saliva of the brown dog tick *Rhipicephalus sanguineus* revealed the presence of chemokine-binding proteins (11–13). These proteins, referred to as evasins, suppress chemokine-driven inflammation by binding and neutralizing multiple chemokines (14). Tick evasins fall into two structurally and functionally unrelated classes. Evasins 1 and 4 (EVA1 and EVA4) are homologous, with eight conserved Cys residues, and specifically bind subsets of CC-chemokines, whereas EVA3 has six Cys residues and specifically binds a subset of ELR^+^ CXC-chemokines. Structural characterization of the EVA1–CCL3 complex indicates that it binds with 1:1 stoichiometry, with the N terminus and N-loop of CCL3 binding the N and C termini of EVA1, with selectivity being determined by the six residues immediately preceding the N-terminal Cys of CCL3 (15). The mechanism underlying the ability of tick evasins to bind multiple specific chemokines in a promiscuous fashion is not understood. Potential mechanisms include differential ligand positioning (16), *i.e.* where different linear segments of the evasin molecule might bind different chemokines, or conformational structural plasticity (17, 18), where the same linear segment may adopt different conformations that enable it to bind different chemokines.

The ability of EVA1, -3, and -4 to potently inhibit inflammation in a wide range of preclinical models provides proof of concept for their use as biological therapeutics in human disease (14) and has stimulated interest in identifying evasin-like molecules from other tick species. We (19–21) and others (22) have recently reported the identification and characterization of several new evasins from diverse tick genera that specifically bind CC- but not CXC-chemokines. Many of these novel evasins have, like EVA1/EVA4, eight conserved Cys residues, and we refer to them as C8 evasins. We have also reported the identification of an EVA3 homolog, P1156, that, like EVA3, binds ELR^+^ CXC-chemokines (21).

Here, we report the identification and functional characterization of 27 novel CXC-chemokine–binding tick evasins. Like EVA3, these novel evasins have six Cys residues, and we refer to them as C6 evasins. We found that the CXC-chemokine–binding C6 evasins can be grouped by function into two classes. Class I, which includes the class founder EVA3, binds ELR^+^ CXC-chemokines, including CXCL1 and/or CXCL8, whereas class II, which is novel, binds a broader range of ELR^+^ and ELR^−^ chemokines but, surprisingly, does not bind CXCL8. We report the X-ray crystal structure of EVA3 and show that it has a disulfide-bonded knottin scaffold. We found that discrete segments within the knottin scaffold form a solvent-accessible contiguous surface and are necessary for specific CXC-chemokine–binding activities. Our results indicate that promiscuous but specific CXC-chemokine recognition by EVA3 and its homologs is, at least in part, mediated by differential ligand positioning on the knottin scaffold and that manipulation of this scaffold surface can be used to engineer evasins with altered properties.

## Results

### Yeast surface display identifies novel CXC-chemokine–binding evasins

We identified 119 putative EVA3 homologs (including the previously reported P1156) by searching publicly available tick salivary transcriptome libraries using three iterations of psiBLAST with the EVA3 sequence as described previously (19–21). 114 of these sequences were from *Ixodes ricinus*, four were from *Amblyomma cajennense*, and one was from *Rhipicephalus pulchellus*. We cloned cDNAs encoding the mature protein sequences from all 119 EVA3 homologs (and EVA3 itself) into three different yeast expression shuttle vectors. The three shuttle vectors were designed to fuse each tick protein with surface display tags either at the N terminus (AGA2) or at the C terminus (AGA2 and SAG1) under the control of an inducible GAL4 promoter. The plasmid library pool was amplified, transformed into yeast as a pool, and induced to express library-encoded proteins on the cell surface with galactose. The pooled yeast library was screened with individual biotinylated chemokines (CXCL1, -7, -8, -9, -10, -11, and -12) followed by streptavidin-AF647 as described previously (19–21). Briefly, labeled yeast cells were sorted by FACS using a sorting gate, which was determined by FACS analysis of the yeast cells labeled with streptavidin-AF647 alone to exclude cells that nonspecifically bound to streptavidin-AF647. Cells recovered were regrown, sorted once more, and then plated at low density, allowing individual yeast clones to be picked and retested to verify binding to the biotinylated chemokine. In these experiments, individual yeast clones were labeled with streptavidin-AF647 alone (control) and biotinylated chemokine plus streptavidin-AF647 (Table S1). Inserts from plasmid DNA extracted from yeast clones that had detectable binding (above the control threshold; Table S1) were amplified by PCR and sequenced to identify the evasin. In selected cases, yeast clones were also compared with control yeast expressing the relevant surface display tag by FACS (Fig. S1) to confirm binding to the screening chemokine.

### Expression and purification of novel evasins in mammalian cells

We successfully expressed EVA3 and the 28 putative evasins identified in the yeast display screen as secreted C-terminal StrepII:His-tagged proteins from HEK293F mammalian cells and purified them using nickel affinity followed by size-exclusion chromatography as described previously (19–21). All purified proteins stained with the periodic acid–Schiff method, indicating that they were glycosylated. They also migrated at molecular weights that were larger than expected, consistent with their predicted and observed glycosylation (Fig. S2).

### EVA3 homologs fall into two distinct classes defined by chemokine-binding activity

We assayed the binding of EVA3 and all the novel evasins to CXC- and CC-chemokines using biolayer interferometry as described previously (19–21). Evasins were bound to a nickel-coated biosensor probe through the C-terminal His tag. We initially screened a panel of human CC- and CXC-chemokines at 300 nm chemokine concentration. None of the evasins bound to CC-, CX3C-, or XC-chemokines. Several evasins that were identified by yeast surface display (*e.g.* those arising from the CXCL9 screen) did not bind the screening chemokine by biolayer interferometry (BLI; Table S1) but bound other CXC-chemokines in the same assay. A single evasin, P1101, which was isolated in the CXCL10 screen, did not bind any chemokine by biolayer interferometry (Fig. S3 and Table S1). The lack of concordance in these instances between yeast surface display and biolayer interferometry, which was performed using proteins produced in mammalian cells, could potentially be explained by known differences in post-translational modifications such as glycosylation and sialylation between yeast and mammalian cells.

**Figure 1.**
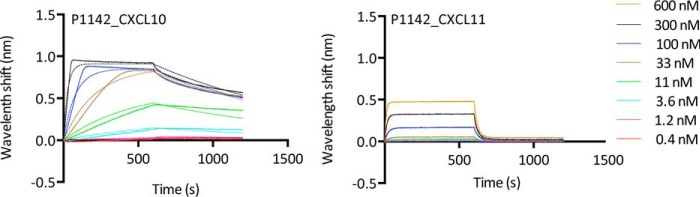
**Characterization of ELR^−^ chemokine binding using biolayer interferometry.** Biolayer interferometry sensorgrams show P1142 binding to different doses of chemokines CXCL10 (*left panel*) and CXCL11 (*right panel*). Plots display wavelength shift (*y axis*; nm) *versus* time (*x axis*; seconds). *Solid lines* indicate collected data; *dashed lines* indicate fitted data.

**Figure 2.**
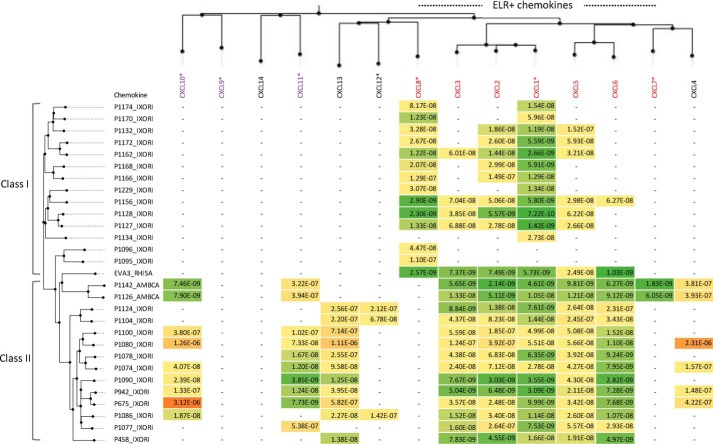
**Summary data of chemokine binding using biolayer interferometry.** Binding affinities (*K_d_*; m) of immobilized evasins to human CXC-chemokines using biolayer interferometry are shown. High-affinity binding is indicated as shades of *green*, medium affinity is indicated as *yellow*, and low affinity is indicated as shades of *orange*. Chemokines and evasins are arranged by sequence similarity–based phylogeny. Inflammatory chemokines are colored either *red* (ELR^+^) or *purple* (ELR^−^). A *dash* (–) indicates that binding was not detected at 300 nm chemokine concentration on the cross-binding screen. An *asterisk* following a chemokine indicates that it was used for yeast surface display screening. Data for P1156_IXORI were reported previously (21) and are shown for comparison. Evasin functional classes I and II are indicated; see text for details.

**Figure 3.**
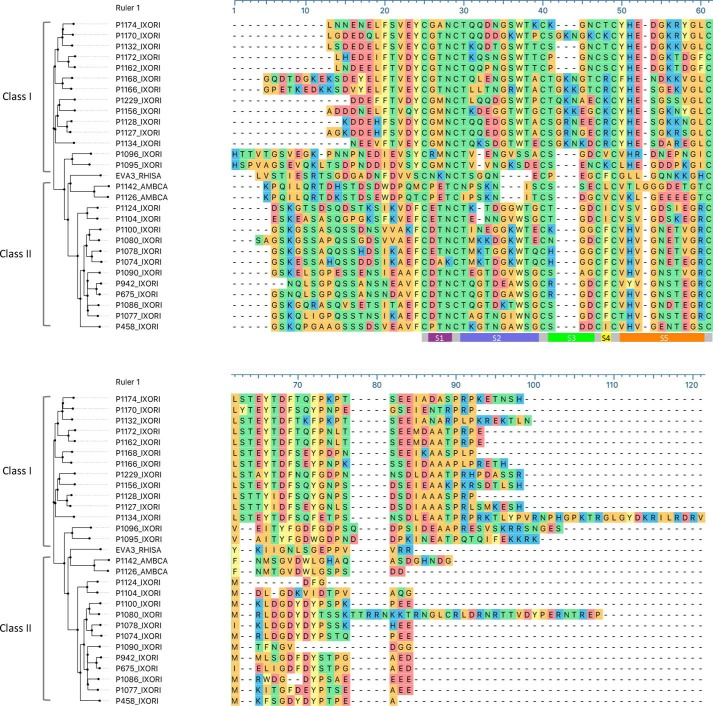
**Analysis of CXC-chemokine–binding evasins.** MUSCLE alignment of CXC-chemokine–binding evasins identified by yeast surface display with EVA3 (*EVA3_RHISA*) is shown. The *gray bar* indicates the conserved disulfide-bonded central core. *Bars* indicating segments S1–S5 between Cys residues are colored as *magenta*, *blue*, *green*, *yellow*, and *orange*, respectively. Sequences are arranged by sequence similarity–based phylogeny. The protein sequence *prefix* indicates the identity, and the *suffix* indicate the tick species as follows: *RHISA*, *R. sanguineus*; *AMBCA*, *A. cajennense*; *IXORI*, *I. ricinus*. Amino acid residues are color-coded by physicochemical properties: *yellow*, aromatic (Phe, Trp, and Tyr); *red*, acidic (Asp and Glu); *blue*, basic (Arg, His, and Lys); *orange*, nonpolar aliphatic (Ala, Gly, Ile, Leu, Met, Pro, and Val); *green*, polar neutral (Cys, Asn, Gln, Thr, and Ser). Evasin functional classes I and II are indicated; see text for details.

We next performed titration assays with serial dilutions of chemokines that bound at 300 nm to determine binding affinities (Figs. 1 and 2). Target residence times calculated from the off-rate are shown in Fig. S4. These studies identified two functional classes of EVA3 homologs. The 15 members of class I, which includes EVA3 and P1156, bind ELR^+^ CXC-chemokines. The 14 members of class II bind both ELR^+^ and ELR^−^ CXC-chemokines (Figs. 1 and 2). All Class I EVA3 homologs bound CXCL8 barring P1134. The 14 novel class II EVA3 homologs, in addition to their ELR^+^-binding activity, bound to one or more of the non-ELR chemokines CXCL10, CXCL11, CXCL13, CXCL12, and CXCL4. Notably, no evasin that bound CXCL8 was found to bind an ELR^−^ chemokine. Two closely related evasins, P1142 and P1126, both from *A. cajennense*, shared high-affinity (*K_d_* < 10 nm) binding to CXCL10 and low-affinity binding (*K_d_* > 300 nm) to CXCL11. Two other evasins, P942 and P675, from *I. ricinus*, had the reverse binding specificity, binding CXCL11 strongly (*K_d_* < 20 nm) and CXCL10 weakly (*K_d_* > 100 nm) (Figs. 2 and 3). We also identified several evasins that bound the homeostatic chemokines CXCL12 and CXCL13. In particular, P1104, from *I. ricinus*, bound to CXCL12. The target residence times of the novel evasins varied from brief (<1 min) to >10 min, *e.g.* for the binding of P1142 to CXCL10, suggesting that in some cases relatively stable evasin–chemokine complexes could form.

### EVA3 homologs contain six conserved Cys residues and are glycosylated

We aligned the protein sequences of the 27 evasins that were confirmed by BLI to bind one or more CXC-chemokines with EVA3 and the previously reported P1156 (Fig. 3). All recovered evasins had six conserved Cys residues, the arrangement being C*X*_3_C*X*_6,10_C*X*_3,6_C*X*_1_C*X*_10,11_C, with subscript numbers indicating spacing between Cys residues. These proteins shared between 26 and 50% identity with EVA3, ranged in length from 61 to 104 residues (molecular mass between 6.1 and 11.9 kDa), exhibited isoelectric points (pI) between 3.69 and 8.51, and typically had one or more predicted *N*- and *O*-linked glycosylation sites (Table S2). Analysis of residue conservation in the alignment (Fig. S5) shows that the N and C termini of these novel evasins are poorly conserved compared with the central core region.

### X-ray structural analysis of EVA3 reveals a disulfide-bonded knottin scaffold

**Figure 4.**
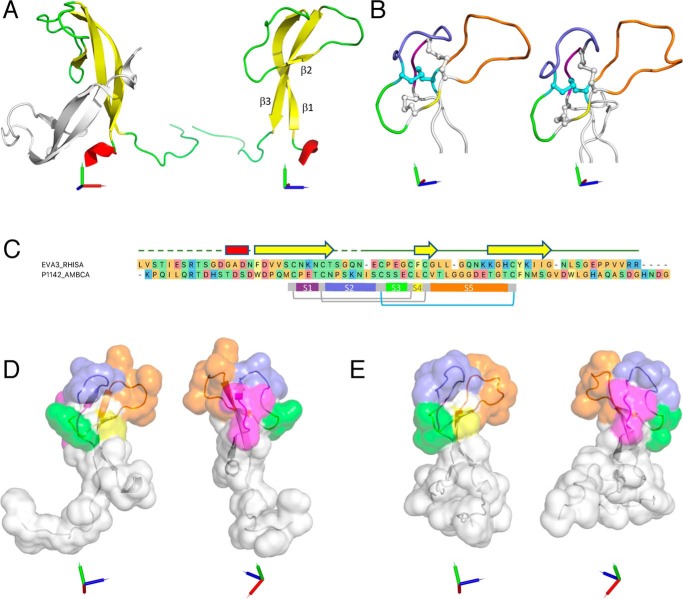
**X-ray crystal structure of EVA3 and model of P1142.** *A*, *left panel*, ribbon structure of EVA3 dimer. Chain A is colored *red* (α-helix), *yellow* (β-strands), and *green* (loops). Chain B is indicated as *gray. Right panel*, ribbon structure of EVA3 chain A rotated through 90° about the *y* axis with the three β-sheets indicated. Orthogonal *x*, *y*, and *z* axes are indicated as *red*, *green*, and *blue*, respectively. *B*, EVA3 chain A (*left*) and P1142 homology model (*right*) showing disulfide bonds between Cys^1^ and Cys^4^ and between Cys^2^ and Cys^5^ (*gray*) that form a macrocycle between segment S1 (*magenta*) and S4 (*yellow*) and the Cys^3^–Cys^6^ disulfide bond (*cyan*) that passes through the macrocycle. Segments S2, S3, and S5 are colored *blue*, *green*, and *orange*, respectively. For clarity, the main chain is shown as a *tube*. Orthogonal *x*, *y*, and *z* axes are indicated as r*ed*, *green*, and *blue*, respectively. *C*, MUSCLE alignment of EVA3 and P1142. Secondary structures in EVA3 chain A are indicated above the EVA3 sequence and are colored as a *red bar* (α-helix), *yellow arrow* (β-strands), and *green line* (loop). The *dashed green line* indicates residues that are not visible in the EVA3 chain A or chain B X-ray crystal structures. Disulfide bonds between Cys^1^ and Cys^4^ and between Cys^2^ and Cys^5^ are indicated in *gray*, and that between Cys^3^ and Cys^6^ is in *cyan*. The *gray bar* indicates the conserved disulfide bonded central core. *Bars* indicating segments *S1–S5* are colored as above. Amino acid residues are color-coded by physicochemical properties: *yellow*, aromatic (Phe, Trp, and Tyr); *red*, acidic (Asp and Glu); *blue*, basic (Arg, His, and Lys); *orange*, nonpolar aliphatic (Ala, Gly, Ile, Leu, Met, Pro, and Val); *green*, polar neutral (Cys, Asn, Gln, Thr, and Ser). *D*, solvent-accessible surface of EVA3 is shown in two orientations with segments S1–S5 colored on the surface as above. Orthogonal *x*, *y*, and *z* axes are indicated as *red*, *green*, and *blue*, respectively. *E*, solvent-accessible surface of P1142 homology model is shown in two orientations with segments S1–S5 colored on the surface as above. Orthogonal *x*, *y*, and *z* axes are indicated as *red*, *green*, and *blue*, respectively.

As a first step to understanding how EVA3 and its homologs bind and neutralize different classes of CXC-chemokines, we determined the X-ray crystal structure of EVA3. EVA3 was expressed and purified from *Escherichia coli* and crystallized readily. The structure was solved essentially by the SIRAS method (see “Experimental procedures”). The crystal structure of the nonglycosylated EVA3 contains two molecules per asymmetric unit, monomer A (Gly^13^–Arg^66^) and monomer B (Asp^12^–Asn^56^) (Fig. 4*A*). The two monomers are very similar, and the Cα of residues 13–56 can be overlaid with a root-mean-square deviation of 1.0 Å. In both monomers, the initial N-terminal residues and the C-terminal end, comprising the six-histidine tag, are flexible and are not seen in the electron density maps. Residues Leu^57^ to the C-terminal Arg^66^ are not visible in monomer B and are only visible in monomer A because of an extensive β-sheet interaction of this region of the protein with a symmetry-related monomer. The EVA3 monomer is composed essentially of a single domain containing a large antiparallel β-sheet composed of residues Phe^17^–Thr^27^ (β1) and residues Lys^47^–Gly^55^ (β3), with residues Phe^38^–Gly^40^ forming a third short additional strand (β2) interacting with residues His^49^–Tyr^51^ of β3. Two extensive loop regions, composed of residues Ser^28^–Cys^37^ and Leu^41^–Lys^47^ join the two major β-strands, whereas a very short α-helix is present at the N-terminal region residues Ala^14^–Asn^16^. The short β2 strand, which interacts with β3, is held in place by a disulfide bridge between residues Cys^26^ and Cys^39^. The first loop, between strands β1 and β2, is rigidified by the presence of two other disulfide bridges between Cys^33^ and Cys^50^ and Cys^37^ and Cys^22^. The topology of EVA3 is that of an archetypal knottin cystine knot (23), with the Cys^3^–Cys^6^ disulfide bond (Cys^33^–Cys^50^ in EVA3) traversing the macrocycle created by Cys^1^–Cys^4^ (Cys^22^–Cys^37^ in EVA3) and Cys^2^–Cys^5^ (Cys^26^–Cys^39^ in EVA3) disulfide bonds (Fig. 4*B*). This was confirmed by examining chain A using the KNOTER3D tool available in the KNOTTIN database (24) (Fig. S6). The crystal structure also contains a cadmium ion present in the crystallization solution that binds to the side-chain oxygen atoms of Glu^60^, carbonyl oxygen of Leu^57^, and carbonyl of Gly^36^ of the symmetry-related chain A molecule.

### The knottin scaffold of EVA3 creates a contiguous solvent-accessible surface

To understand the mechanism of chemokine recognition by the two different C6 evasin classes, we focused on P1142 and EVA3. P1142 binds CXCL10 with high affinity but does not bind CXCL8, whereas EVA3 binds CXCL8 with high affinity but does not bind CXCL10 (see Fig. 2). In the absence of a structure for P1142, we used the EVA3 chain A template to model full-length P1142 using the program MODELLER (25). The models (Figs. 4, *B* and *C*, and S7) suggested that P1142 has a disulfide-bonded core structure similar to EVA3. Modeling of the solvent-accessible surfaces of EVA3 chain A and of the P1142 model indicated that five predicted solvent-exposed segments, S1 between Cys^1^ and Cys^2^, S2 between Cys^2^ and Cys^3^, S3 between Cys^3^ and Cys^4^, S4 between Cys^4^ and Cys^5^, and S5 between Cys^5^ and Cys^6^, are created by the disulfide-bonded knottin scaffold. These segments (referred to as “loops” in the knottin literature (23)) are predicted to form a contiguous solvent-accessible surface, with the conserved cysteine residues buried within the core, in both EVA3 and P1142 (Figs. 4, *D* and *E*, and S6). The primary structure of these segments is poorly conserved between EVA3 and P1142 (Fig. 4*C*).

### Distinct knottin scaffold segments bind different CXC-chemokines

**Figure 5.**
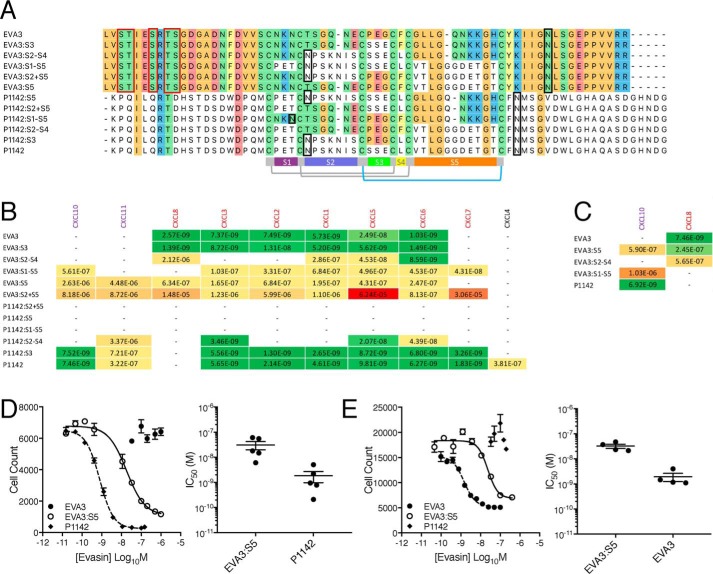
**Analysis of chemokine binding by EVA3 and P1142.** *A*, CLUSTAL alignment of EVA3 and P1142 hybrids. Amino acid residues identical to EVA3 are color-coded by physicochemical properties: *yellow*, aromatic (Phe, Trp, and Tyr); *red*, acidic (Asp and Glu); *blue*, basic (Arg, His, and Lys); *orange*, nonpolar aliphatic (Ala, Gly, Ile, Leu, Met, Pro, and Val); *green*, polar neutral (Cys, Asn, Gln, Thr, and Ser). Disulfide bonds between Cys^1^ and Cys^4^ and between Cys^2^ and Cys^5^ are indicated in *gray*, and that between Cys^3^ and Cys^6^ is in *cyan*. The *gray bar* indicates the disulfide-bonded central core. The intercysteine segments are indicated by *colored bars*: *magenta* (S1), *blue* (S2), *green* (S3), *black* (S4), and *orange* (S5). *Red* and *black boxes* around residues indicate predicted *O*-glycosylation and *N*-glycosylation sites, respectively. *B*, binding affinities (*K_d_*; m) of immobilized parental evasins and hybrids to human CXC-chemokines using biolayer interferometry. High-affinity binding is indicated as shades of *green*, medium affinity is indicated as *yellow*, and low affinity is indicated as shades of *orange*. Inflammatory chemokines are colored either *red* (ELR^+^) or *purple* (ELR^−^). A *dash* (–) indicates that a binding constant could not be calculated. Data in *italics* indicate results where only four data points could be fitted. *C*, binding affinities (*K_d_*; m) of immobilized parental evasins and hybrids to human CXC-chemokines using biolayer interferometry. BLI was performed as described under “Experimental procedures” except that seven chemokine doses ranging from 1000 to 0.8 nm were used. High-affinity binding is indicated as shades of *green*, medium affinity is indicated as *yellow*, and low affinity is indicated as shades of *orange*. Inflammatory chemokines are colored either *red* (ELR^+^) or *purple* (ELR^−^). A *dash* (–) indicates that a binding constant could not be calculated and is interpreted as lack of binding. *D*, neutralization of human CXCL10-induced T-lymphocyte migration. The *left panel* shows a representative experiment of CXCL10-induced T-lymphocyte migration by EVA3:S5, P1142, and EVA3, respectively. The *y axis* shows cell count of T-cells migrating through to the bottom chamber in response to an EC_80_ dose of CXCL10. Data are shown as mean ± S.E. of three technical replicates. The *x axis* shows evasin concentration (log_10_ molar). IC_50_ values (m) indicated in each figure were estimated by fitting an agonist-response curve with four parameters. The *right panel* shows individual IC_50_ values and mean ± S.E. of five biological replicates. Mean IC_50_ for EVA3:S5 is 3.11 × 10^−8^ m, whereas that for WT P1142 is 1.85 × 10^−^9 m. No inhibition by EVA3 was observed. *E*, neutralization of human CXCL8-induced granulocyte migration. The *left panel* shows a representative experiment of CXCL8-induced granulocyte migration by EVA3:S5, EVA3, and P1142, respectively. The *y axis* shows cell count of granulocytes migrating through to the bottom chamber in response to an EC_80_ dose of CXCL8. Data are shown as mean ± S.E. of three technical replicates. The *x axis* shows evasin concentration (log_10_ molar). IC_50_ values (m) indicated in each figure were estimated by fitting an agonist-response curve with four parameters. The *right panel* shows individual IC_50_ values and mean ± S.E. of four biological replicates. Mean IC_50_ for EVA3:S5 is 3.23 × 10^−8^ m, whereas that for WT EVA3 is 1.94 × 10^−^9 m. No inhibition by P1142 was observed. *Error bars* represent S.E.

To explore the role of the predicted solvent-exposed surface created by the disulfide-bonded scaffold, we constructed and produced hybrid proteins that swapped one or more of the solvent-exposed segments between EVA3 and P1142 (Fig. 5, *A* and *B*). These hybrid proteins were expressed in the mammalian expression system as described above (Fig. S8). First, the small S3 segment in EVA3 was replaced with the equivalent segment in P1142 (construct EVA3:S3) and vice versa (construct P1142:S3). The S3 variants displayed similar binding affinities to CXCL8 or CXCL10 as the parental evasins (Fig. 5*B*), showing that the S3 segment does not confer specificity in binding to CXCL8 or CXCL10. However, it does appear to be required for binding to CXCL4 in P1142. Next, a larger region between Cys^2^ and Cys^5^, containing segments S2, S3, and S4, was swapped between the evasins (S2–S4 variants). P1142:S2–S4 lost CXCL10, CXCL2, CXCL1, and CXCL7 binding, indicating that S2-S3-S4 was required for binding to these chemokines. There was a reduction in binding to CXCL5 and -6, indicating that this region in P1142 was necessary for binding these chemokines and that the equivalent region of EVA3 could not substitute. EVA3:S2–S4 displayed a marked reduction in affinity toward CXCL8 compared with EVA3 or EVA3:S3. The reduction in binding affinity suggests that the S2 and S4 segments of EVA3 contribute to the interaction with CXCL8. In addition, there was a loss of binding to CXCL2 and -3, indicating that S2 and S4 segments were required for binding to these chemokines and could not be substituted by the equivalent segments of P1142.

All five segments (S1–S5) were next swapped between the evasins (S1–S5 variants). Notably, CXCL10 binding was now partially transferred to EVA3:S1–S5, and CXCL8 binding was lost (Figs. 5*B* and S8). Taken together with the P1142:S2–S4 result above, this indicates that a likely binding site for CXCL10 lies either in the S5 and/or in the S1 segment of P1142, in addition to S2/S4. Likewise, it indicates that binding sites for CXCL8 lie either in the S5 and/or in the S1 segment of EVA3, in addition to the S2 and S4 segments identified above. No binding constants could be determined for the chemokine interactions of P1142:S1–S5. This may in part be due to the predicted introduction of a nonnative *N*-linked glycosylation motif at position Asn^24^ that may interfere with protein–protein interactions or protein folding (Fig. 5*A*). Taken together, the analysis of the hybrid proteins suggests that the S1 and S5 segments of the disulfide-bonded scaffold confer specificity in CXCL8/CXCL10 binding, whereas S2 and S4 segments are also needed for binding. Moreover, they indicate that certain chemokines (*i.e.* CXCL1, CXCL2, CXCL3, CXCL5, and CXCL6) also likely bind to the parental evasins by distinctive mechanisms mediated by segments of the disulfide-bonded scaffold, as binding is lost or reduced in the hybrid evasins.

To further dissect the mechanism of binding, segments S2 and S5 or S5 alone was swapped between the evasins (S2+S5 and S5 variants). Intriguingly, the EVA3:S2+S5 and EVA3:S5 variant displayed binding to both CXCL8 and CXCL10 (Figs. 5*B* and S8). Binding affinities could not be determined for P1142:S2+S5 or P1142:S5. The reason is unclear but may in part be due to effects that may interfere with protein–protein interactions or protein folding. The dual CXCL8- and CXCL10-binding properties of EVA3:S5 were confirmed by conducting BLI experiments in parallel with EVA3 and P1142 using a wider concentration range of chemokine (Figs. 5*C* and S8).

We next examined the ability of EVA3:S5 to neutralize CXCL10 and CXCL8. We found that EVA3:S5 would neutralize the ability of CXCL10 to recruit activated T-cells, whereas EVA3 was unable to do so (Fig. 5*D*). EVA3:S5 retained the ability to inhibit CXCL8-mediated granulocyte recruitment (Fig. 5*E*), consistent with the BLI data. These experiments indicate that the 11 amino acids comprising the S5 segment of P1142 confer specificity in CXCL10 binding and neutralization that can be transferred to EVA3.

### Differences in shape and charge potentially explain distinctive binding activities

**Figure 6.**
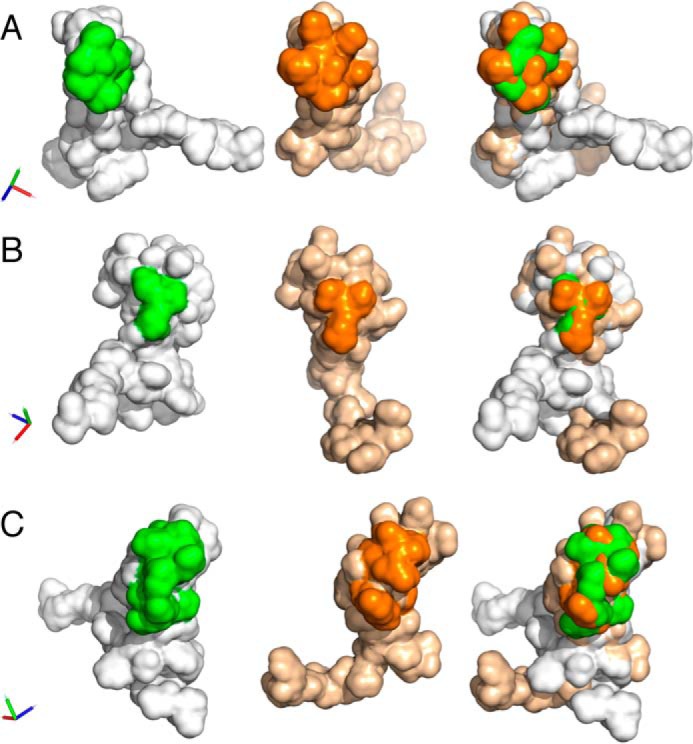
**Shape of P1142 and EVA3 solvent-accessible surfaces.** *A–C*, solvent-accessible surfaces of P1142 and EVA3 highlighting segments S5 (*A*), S1 (*B*), S2-S3-S4 (*C*), respectively. *Left panels*, P1142 (*gray*, with indicated segment in ^green^); *middle panels*, EVA3 (*wheat*, with indicated segment in *orange*), and *right panels*, overlaid aligned molecules. In each panel, the orthogonal *x*, *y*, and *z* axes are indicated as *red*, *green*, and *blue*, respectively.

**Figure 7.**
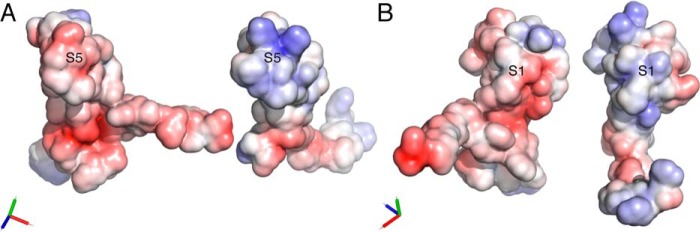
**Charge of P1142 and EVA3 solvent-accessible surfaces.** *A*, 3D structural models of P1142 (*left*) and EVA3 (*right*) showing solvent-accessible surface with surface electrostatic potential indicated as *red* (negative charge) and *blue* (positive charge). The S5 segment is indicated. *B*, 3D structural models of P1142 (*left*) and EVA3 (*right*) with surface electrostatic potential displayed as above, showing the S1 segment. Orthogonal *x*, *y*, and *z* axes are indicated as *red*, *green*, and *blue*, respectively.

Taken together, the above studies identified three segments of the P1142 and EVA3 disulfide-bonded scaffold that likely play an important role in CXC-chemokine binding. These are (*a*) the S5 region of P1142, which can be transplanted to EVA3, conferring ability to bind CXCL10 and CXCL11; (*b*) the P1142 S2-S3-S4 region, which is important for binding CXCL10, CXCL2, CXCL1, CXCL4, and CXCL7 as it cannot be substituted by the equivalent region of EVA3; and (*c*) the S1 region of EVA3, which is necessary for CXCL8 binding as swaps where this is replaced by the P1142 S1 region no longer bind CXCL8. Complementarity in surface shape and charge play important roles in directing the specificity of protein–protein interactions (26, 27). Analysis of solvent-accessible surface shapes of the above regions in aligned models of EVA3 and P1142 indicates substantial differences between the shapes of S5 (Fig. 6*A*), S1 (Fig. 6*B*), and S2-S3-S4 (Fig. 6*C*). Analysis of the surface charge of EVA3 and P1142 solvent-accessible surfaces (Fig. 7, *A* and *B*) indicates that the S5 and S1 segments in P1142 are negatively charged, whereas the corresponding segments of EVA3 are positively charged. The positive charge in EVA3 is contributed by lysine residues, and the negative charge in P1142 is contributed by aspartate and glutamate residues. Analysis of the S5 segment in all CXCL8-binding evasins shows that there is a cluster of positively charged lysine or arginine residues (see Fig. 3). Conversely, the S5 and/or S1 segments contains negatively charged aspartate or glutamate residues in all CXCL10-binding evasins (see Fig. 3). Taken together, these results suggest that the differences in shape and charge likely contribute to the binding of CXCL8 and CXCL10 by EVA3 and by P1142, respectively, and, by extension, to the two different C6 evasin classes.

## Discussion

The major findings that emerge from these studies are, first, the discovery and characterization of novel EVA3 homologs that bind specific CXC-chemokines in a promiscuous fashion and, second, the identification of two functional classes of EVA3 homologs that bind either only ELR^+^ or both ELR^+^ and ELR^−^ chemokines. This includes in some cases the ability to bind CXCL10, a major player in Th1-mediated immunity. Finally, we report the first X-ray crystal structure of the CXC-chemokine–binding evasin EVA3 and the discovery of a novel disulfide-bonded knottin scaffold that underlies the remarkable ability of EVA3 homologs to engage in promiscuous but specific CXC-chemokine binding.

Using yeast surface display, to date, we have identified, including the previously reported P1156 (21), 28 novel CXC-chemokine–binding proteins that share a six-cysteine-residue architecture resembling EVA3. These EVA3-like proteins have been confirmed to bind CXC-chemokines following purification in mammalian expression systems. Notably, only a subset of CXC-chemokines was used in the yeast surface display screens, and it is possible that yet more CXC-chemokine–binding evasins exist. Remarkably, 26 of these CXC-chemokine–binding evasins are from the castor bean tick *I. ricinus*, and only two from the Cayenne tick *A. cajennense*. This bias most likely reflects the original population of the yeast surface display library, which consisted predominantly of *I. ricinus* sequences. Our unpublished psiBLAST analysis of the *I. ricinus* salivary transcriptome identified 147 putative evasin homologs of which 114 were putative EVA3 homologs, predicted to bind CXC-chemokines; the rest were putative EVA4/EVA1 homologs that would be predicted to bind CC-chemokines. By comparison, our unpublished analysis of the *A. cajennense* salivary transcriptome revealed 72 putative evasin homologs of which 68 were EVA4/EVA1 homologs and four were EVA3 homologs. Our previous studies have identified several CC-chemokine–binding EVA4/EVA1-like evasins from *Amblyomma* but none from *Ixodes* (19–21). This suggests that *I. ricinus* and *Amblyomma* species, which represent prostriate and metastriate tick lineages, respectively, that diverged ∼241 million years ago (28), evolved distinctive strategies to target the chemokine network. These observations, together with direct studies of anticytokine activity in tick saliva (29), suggest that *Ixodes* predominantly targets CXC-chemokines, whereas *Amblyomma* targets both CC- and CXC-chemokines.

Like other evasins (15, 19, 30), these CXC-chemokine–binding evasins are glycosylated when expressed in mammalian cells and have several predicted *N*- and *O*-linked glycosylation sites. The glycosylation is consistent with the presence of several molecular weight species on size-exclusion chromatography and slower migration on electrophoresis. The function of glycosylation may be to reduce immunogenicity (31, 32) and enhance protein stability (33). It remains to be shown whether evasin glycosylation directly affects interactions between all evasins and chemokines. In the case of EVA1, glycosylated and deglycosylated versions have similar binding properties, and the structures of both forms are similar (15). EVA3 was similarly equally active in both forms. However, we have found that deglycosylated P672 has a substantially reduced affinity for its target, CCL8 (20).

A clear finding emerging from our analyses is that CXC-chemokine–binding evasins fall into two distinct classes. Members of the first class, which includes the class founder EVA3, bind one or more ELR^+^ CXC-chemokines, including CXCL1 and/or CXCL8, but not ELR^−^ chemokines. Members of the second class bind both ELR^+^ and ELR^−^ chemokines but, surprisingly, do not bind CXCL8. Both CXC-chemokine–binding evasin classes are found in *I. ricinus*. As a major function of ELR^+^ chemokines is to recruit neutrophils via CXCR1/2 binding (2), the significance of ELR^+^ CXC-chemokine binding, we suggest, would be to inhibit the early neutrophilic infiltration that occurs at the site of the tick bite (34). CXCL10 and -11 play important roles in Th1-mediated immunity (2, 35), and inhibition of these chemokines by several ELR^−^ CXC-chemokine–binding evasins may mediate, in part, the suppression of Th1 responses observed in tick infestation (34). We observed that several evasins that display strong binding to CXCL11 only weakly bind CXCL10 and vice versa. CXCL10 and CXCL11 have been suggested to have opposite activities (36) with CXCL10 promoting and CXCL11 suppressing T-cell–mediated autoimmunity. Thus, evasins that preferentially bind CXCL10 may be useful tools to modulate T-cell–mediated autoimmunity.

Our X-ray crystallographic and homology modeling studies indicate that EVA3 and its homologs have a topology referred to as a cystine knot (23) and belong to the class of proteins known as knottins. The disulfide-bonding pattern (but not the cystine knot topology) of EVA3 was recently predicted using selenocysteine scanning (37), and our X-ray crystallographic analyses are consistent with this previous report. Knottins form the largest inhibitor cystine-knot subgroup and are characterized by the knot-forming disulfide bond between Cys^3^ and Cys^6^ traversing a macrocycle created by disulfide bonds between Cys^1^ and Cys^4^ and between Cys^2^ and Cys^5^. Other subgroups of inhibitor cystine knots are cyclotides, where the backbone is also cyclized, and growth factor cystine knots, which have a different topology. Knottins are widely found in eukaryotes, with molecular activities ranging from ion channel blockade to protease inhibition. The cystine-knot structure provides a high degree of stability, maintaining both structure and function following exposure to proteolytic enzymes, pH change, and temperature variation, potentially allowing oral delivery (23, 38). The chemical diversity created from variation in the loop segments that mediate protein interactions and the ability to readily engineer the loop segments render these molecules attractive for development as therapeutic and diagnostic agents (23, 38). Knottins already approved for therapeutic use include linaclotide (Linzess®) and ziconotide (Prialt®). Searches of the online KNOTTIN database (24) suggest that EVA3 and its homologs represent the first structurally documented knottin family isolated from ticks and the first description of knottins that target chemokines, extending the therapeutic potential of the knottin family.

Taken together with the previous report on the structure of EVA1 (15), these results indicate that ticks produce two structurally unrelated chemokine-binding protein classes that each contain novel structural elements. Ticks are arthropods of the subphylum Chelicerata, and importantly, other chelicerate arthropods, spiders and scorpions, have several knottin peptides as venom constituents (39), suggesting that EVA3-like evasins may have originated in the ancestral chelicerate. Molecular analysis indicates that ticks diverged from mites ∼336 million years ago, soon after the appearance of amphibians (28), which may represent the first hosts of ticks (40). CC- and CXC-chemokines are present in amphibians, reptiles, birds, and mammals (41). The appearance of CC- and CXC-chemokine–binding evasins in ticks, which parasitize these vertebrates (40), likely represents an evolutionary adaptation to neutralizing host inflammatory responses, allowing prolonged periods of blood sucking.

Several class I and class II CXC-chemokine–binding evasins possess the remarkable ability to bind multiple specific chemokines in a promiscuous fashion. The mechanisms underlying this are not understood but may include differential ligand positioning (16), *i.e.* where different linear segments of the evasin molecule bind different chemokines, or conformational structural plasticity (17, 18), where the same linear segment adopts different conformations that enable it to bind different chemokines. To investigate this further, we attempted to crystallize EVA3 in complex with a target chemokine, CXCL8. Unfortunately, although EVA3 could be readily crystallized, cocrystals with CXCL8 could not be obtained. Comparison with the structure of EVA1 indicated that there is no similarity between the two tick evasin classes (15). Thus, there was no opportunity to use the EVA1–CCL3 cocrystal structure as a template to model the interaction of EVA3 or its homologs with target CXC-chemokines. We therefore exploited the clear difference in CXCL8- and CXCL10-chemokine binding between EVA3, which binds CXCL8 but not CXCL10, and P1142, which binds CXCL10 but not CXCL8, to explore the binding mechanisms using segment-swapping analyses. These experiments show that discrete solvent-accessible segments within the knottin scaffold surface are important for chemokine binding. Analyses of the surfaces of EVA3 and P1142 suggest that the surfaces created by these segments differ in shape and in charge, which potentially explains the different binding characteristics. Notably, the S5 and/or S1 segment in all CXCL8-binding evasins have a cluster of positively charged residues, whereas the corresponding segments in evasins that bind CXCL10 have negatively charged glutamate or aspartate residues, suggesting that surface charge may be an important factor determining binding specificity. An important caveat to these conclusions is the limited accuracy of prediction of loop structure by homology modeling programs (42). Key questions that need to be addressed are whether the segments identified directly bind to target chemokines independently of the knottin scaffold and the identity of specific residues that mediate such interactions.

In summary, our studies suggest that the ability of the C6 family of evasin proteins to promiscuously bind multiple specific CXC-chemokines arises, at least in part, by differential ligand positioning within the knottin scaffold wherein discrete segments confer different evasin-binding activities. Our studies show that manipulation of the evasin knottin scaffold can create novel proteins with altered chemokine-binding specificities, suggesting the versatility of this scaffold for engineering evasins with desirable chemokine-binding profiles for therapeutic application. Taken together, these studies provide structural and biochemical insight into how CXC-chemokine–binding tick evasins achieve class specificity but are able to engage in promiscuous binding. Importantly, understanding the molecular basis of the promiscuous but specific interactions of the evasins with target chemokines could allow the development of novel knottin-based therapeutics with similar promiscuous mechanisms of action capable of therapeutically tackling complex disease-causing protein-interaction networks.

## Experimental procedures

### Yeast surface display

A library containing 119 putative 6C tick evasins (and also EVA3) was constructed by cloning DNA sequences encoding the mature peptides into yeast surface display plasmids as described previously (19). Yeast surface display screening was performed as described previously using biotinylated CXCL8, CXCL10, CXCL11, and CXCL12 from Almac and biotinylated CXCL1, CXCL7, and CXCL9 produced in house. Briefly, EBY100 yeast transformed with library plasmids were plated on solid medium. Colonies were pooled. The pooled library was grown in liquid culture and induced with galactose to drive expression of the surface-displayed protein. Yeast were labeled with biotinylated chemokines and streptavidin-AF647 and then sorted using FACS. A sorting gate, determined by incubating yeast library with streptavidin-AF647 alone, was used to exclude cells nonspecifically binding streptavidin-AF647. The sorted cells were collected and plated on solid media, and a second round of sorting was performed as above. Following this the cells were plated at low dilution to enable picking of individual colonies. Individual yeast colonies were retested as above to confirm chemokine binding by FACS. Inserts from plasmids isolated from individual colonies were then amplified by PCR and sequenced to identify the cloned evasins.

### Plasmids

Expression plasmids were constructed using idempotent parts using our adaptation of the GoldenGate/GoldenBraid cloning method (19). Yeast plasmids were created in three configurations as described previously (19) with the evasin mature peptide linked to either N-terminal AGA2_YEAST, C-terminal AGA2_YEAST, or C-terminal SAG1_YEAST surface display tags and were driven by a yeast GAL1 promoter. For mammalian expression plasmids, parts included the CAGGS promoter (from GenBank^TM^ accession number AB281497), Igκ signal peptide (43), StrepII:His tag (GGASAWSHPQFEKLEHHHHHHHH, pQE-Trisystem, Qiagen), and bovine growth hormone terminator (pcDNA3, Invitrogen), and sequences encoding the evasin mature peptide were identified in the yeast surface display screen. Following signal peptide cleavage, the expected sequence at the N terminus is DGG, predicted by SignalP 3.0 (44). For bacterial expression plasmids, parts included a modified *E. coli*_lacI:T7:lacO:RBS promoter (from pET28, Novagen), a His_12_-SUMO tag, an Avitag (45), and *E. coli birA* (from Addgene 20856, pDisplay-BirA-ER). The mature peptide encoding sequences of CXCL1 (Ala^35^–Asn^107^), CXCL7 (Ala^59^–Asp^128^), and CXCL9 (Thr^23^–Thr^125^) were cloned in-frame following the SUMO tag such that the N terminus would be correctly created following SUMO protease cleavage. Plasmids, sequences, and detailed methods of construction are available on request.

### Protein sequence analysis

Sequences were aligned using MUSCLE or CLUSTAL in Megalign Pro (DNAStar version 12.3.1, DNAStar Inc.) to construct alignments and generate sequence similarity–based phylogenetic trees as described previously (19). The sequence alignment was analyzed using plotcon in the EMBOSS suite (46) (http://www.bioinformatics.nl/emboss-explorer/).^8^ Glycosylation site prediction was performed using NetNGlyc1.0 (http://www.cbs.dtu.dk/services/NetNGlyc/)^8^ and NetOGlyc4.0 (http://www.cbs.dtu.dk/services/NetOGlyc/)^8^ (62) as described previously (19). Protein molecular weight and pI were calculated at ExPASy (http://web.expasy.org/compute_pi/).^8^

### Evasin production

Evasins (including EVA3) for biolayer interferometry and cell migration assays were produced in mammalian HEK293F cells as described previously (19). Transiently transfected HEK293F cell culture supernatants were loaded on nickel-charged IMAC Sepharose 6 Fast Flow resin (GE Healthcare) followed by size-exclusion chromatography. Fractions demonstrating absorption at 280 nm were analyzed electrophoretically and pooled for subsequent experiments. Evasin glycosylation was analyzed by periodic acid–Schiff staining (786-254, G-Biosciences). EVA3 for crystallization was produced in *E. coli* as described below.

### Chemokine production

Biotinylated chemokines used for yeast surface display screening were expressed in *E. coli*, cleaved with SUMO protease, and purified as described (20). Briefly, *E. coli* RosettaGami^TM^ 2 (DE3) cells (Novagen) were transformed with the relevant expression plasmids and induced with isopropyl 1-thio-β-d-galactopyranoside, and the soluble fraction isolated from the cell pellet by sonication and centrifugation was purified using nickel-charged IMAC Sepharose 6 Fast Flow resin, eluted, and then treated with SUMO protease (a gift from Dr. Ritika Sethi). The cleaved protein was purified by cation-exchange chromatography on a HiTrap Capto S 1-ml cation-exchange column (GE Healthcare) followed by size-exclusion chromatography.

### Biolayer interferometry

Biolayer interferometry was performed on an OctetRed® system as described previously (19). Cross-binding against a panel of human chemokines was performed at 300 nm chemokine concentration. CCL25, CCL26, and CXCL16, which nonspecifically bound to the sensor, were excluded, as were CXCL17, CXCL4L1, and XCL2, which were not available. To assure functionality of different assay components, biolayer interferometry experiments were performed such that all chemokines studied were run in parallel for a given evasin, and several evasins were run in parallel for the chemokine panel. Binding kinetics were evaluated using chemokine concentrations ranging from 600 to 0.4 nm (except where indicated in figure legends) and a noninteracting reference protein to allow for nonspecific binding. ForteBio Data Analysis 9 software was used to process the data and calculate association (*k*_on_), dissociation (*k*_off_), and affinity (*K_d_*) constants. We used all data where the fit *R*^2^ was ≥0.96, and at least five data points were used to calculate the fit. The dissociation half-life or target residence time was calculated as described (19, 47) from biolayer interferometry off-rates (*k*_off_, s^−1^, as *t*_½_ = 0.693/(*k*_off_ × 60)). For the domain-swap hybrid evasins (Fig. 6*B*), due to weaker binding of some of the hybrid mutants, the fitting criterion was adjusted to *R*^2^ ≥ 0.89, and at least five concentration data points were used to calculate the fit except for P1142:S3 and P1142:S2-S4 against CXCL11 and EVA:S2-S4 against CXCL1 and CXCL8,where the *K_d_* fitting was based on four concentrations.

### Cell lines

HEK293F cells were a gift from Nicola Burgess-Brown (University of Oxford). THP-1 cells were purchased from Sigma. A MycoAlert^TM^ (Lonza) kit was used to confirm freedom from *Mycoplasma* contamination. Cells were functionally authenticated by protein production and chemokine-induced migration as appropriate.

### Activated human T-cell migration assay

Peripheral blood mononuclear cells were isolated from human buffy coat by density gradient centrifugation using Lymphoprep^TM^ density gradient medium (STEMCELL Technologies) and centrifuged in SepMate^TM^ isolation tube (85450, STEMCELL Technologies). Human T-cells were further isolated using the human CD8^+^ T-cell isolation kit (07801, STEMCELL Technologies). The enrichment of CD8^+^ T-cells was confirmed by labeling cells with CD8-FITC (130-110-667, Miltenyi Biotec) and CXCR3-APC (130-101-378, Miltenyi Biotec) and analyzed on an Attune NxT flow cytometer. Isolated T-cells were then activated in ImmunoCult^TM^-XF T-cell expansion medium (10982, STEMCELL Technologies) with CD3/CD28 T-cell activator at 25 μl/ml (10991, STEMCELL Technologies) and supplemented with 30 units/ml recombinant human IL-2 (200-02, PeproTech). During expansion, T-cells were passaged every 2–3 days in TexMACS medium (130-097-196, Miltenyi Biotec) with 1% penicillin/streptomycin + 30 units/ml human IL-2 and incubated at 37 °C in 5% CO_2_. Activated T-cells were used 10 days after initial activation and for a further 5 days thereafter, while still proliferating, during which activated CD8^+^ T-cells were maintained at 0.3 × 10^6^ cells/ml until use. The homogeneity of CD8^+^ T-cells was confirmed by labeling with CD8-FITC and CXCR3-APC at days 7 and 10. Human activated T-cell migration assays were performed using 96-well Transwell migration plates (3-μm pore size; 3385, Corning). Cells were counted on an Attune flow cytometer using a forward scatter (FSC) *versus* side scatter (SSC) dot plot and a previously defined gate setting for activated CD8^+^ T-cells. Effective concentrations (EC), EC_80_ and EC_50_, for CXCL10 were determined each day (see Fig. S9). CXCL10 (0–150 nm; PeproTech) in 150 μl of RPMI 1640 medium + 2 mm l-Glu + 0.5% heat-treated fetal bovine serum (all from Sigma) were placed in the bottom chamber. Cells (6 × 10^4^ in 50 μl of RPMI 1640 medium + 2 mm l-Glu + 0.5% heat-treated fetal bovine serum) were placed in the top chamber and incubated at 37 °C in 5% CO_2_ for 2 h. The migration plate was shaken at 850 rpm for 10 min, and medium from the bottom plate was transferred to a U-bottomed 96-well plate. Cells were counted on an Attune flow cytometer using an FSC *versus* SSC dot plot, and data were analyzed in GraphPad Prism, fitting an agonist-response curve with four parameters. IC_50_ values for EVA3:S5 and P1142 were determined using the above system. CXCL10 (EC_80_ dose) and EVA3:S5 (0.015–1000 nm concentration) or P1142 (0.015 nm-150 nm concentration) were added to the bottom chamber and incubated for 30 min at 37 °C before beginning the cell migration assay. Data (three technical replicates for each IC_50_ determination) were analyzed in GraphPad Prism, fitting an inhibitor-response curve with four parameters. The mean IC_50_ from five biological replicates was then calculated.

### Human buffy coat granulocyte migration assay

Human buffy coat sample was collected on the day of experiment. To extract granulocytes, red blood cells were first depleted using HetaSep^TM^ (07906, STEMCELL Technologies). All nucleated cells were then collected, and any remaining red blood cells were lysed using Red Blood Cell Lysis Solution (130-094-183, Miltenyi Biotec). Human granulocyte migration assays were performed using 96-well Transwell migration plates (3-μm pore size). Cells were counted on an Attune flow cytometer using a FSC *versus* SSC dot plot. EC_80_ and EC_50_ for each chemokine were determined each day for each batch of cells isolated from the buffy coat in three technical replicates (see Fig. S9). Chemokines (0–150 nm (PeproTech) in 150 μl of RPMI 1640 medium + 2 mm l-Glu + 0.5% heat-treated fetal bovine serum (all from Sigma) were placed in the bottom chamber. Cells (2 × 10^5^ in 50 μl of RPMI 1640 medium + 2 mm l-Glu + 0.5% heat-treated fetal bovine serum) were placed in the top chamber and incubated at 37 °C in 5% CO_2_ for 1 h. The migration plate was shaken at 850 rpm for 10 min, and medium from the bottom plate was transferred to a U-bottomed 96-well plate. Cells were counted on an Attune flow cytometer using an FSC *versus* SSC dot plot, and data were analyzed in GraphPad Prism, fitting an agonist-response curve with four parameters. IC_50_ values for EVA3:S5 and EVA3 were determined using the above system. CXCL8 (EC_80_ dose) and EVA3:S5 (0.05–200 nm concentration), EVA3 (0.02 nm-100 nm concentration), or P1142 (100 nm) were added to the bottom chamber and incubated for 30 min at 37 °C before beginning the cell migration assay. Data (three technical replicates for each IC_50_ determination) were analyzed in GraphPad Prism, fitting an inhibitor-response curve with four parameters. The mean IC_50_ from four biological replicates was then calculated.

### Crystallization and structure determination of EVA3

EVA3 was expressed from a pET30a expression vector in *E. coli* strain BL21(DE3). The protein was purified from the soluble cytosolic fraction by cation-exchange chromatography on Fractogel-SO_3_ in 50 mm sodium acetate, pH 4.5, by a linear gradient of 0–0.7 m NaCl followed by size-exclusion chromatography on an Superdex 75 16/60 column (GE Healthcare) in PBS. The purified protein was dialyzed against 50 mm NH_4_HCO_3_, aliquoted, lyophilized, and stored at −20 °C. The first crystals of evasin 3 produced in *E. coli* were obtained from sitting-drop vapor-diffusion screening using assorted incomplete factorial screens. The most suitable crystals of EVA3 were obtained by vapor diffusion by adding 2 μl of protein solution at 6 mg/ml in 50 mm Tris-HCl, pH 8.0, containing 100 mm NaCl to 1 μl of 100 mm CdCl_2_ and 2 μl of the well solution, which was composed of 25% PEG 3350 (v/v) and 100 mm Bis-Tris buffer at pH 6.5. The crystals were briefly transferred to a solution composed of the well solution supplemented with 15% (v/v) glycerol, and the crystals were frozen at 100 K. The crystals diffract beyond 1.80 Å at a synchrotron source, belong to the space group P3_1_21 with unit-cell dimensions of *a* = *b* = 55.09 Å and *c* = 71.04 Å, have a calculated solvent content of ∼45%, and were expected to contain two molecules of evasin 3 per asymmetric unit. Two potential heavy-atom-derivative soaks were prepared by soaking the crystals for 24 h in well solution supplemented with 1 mm K_2_PtCl_4_ or 1 mm AuKCl_4_. All data sets were collected at the X06SA-PXI beamline of the Swiss Light Source (SLS) at the Paul Scherrer Institute (PSI) in Villigen, Switzerland. Data were indexed and processed using MOSFILM (48) and SCALA from the CCP4 package (49). Initial heavy-atom positions were determined by Patterson methods using SHELXL (50) and further refined with SHARP (51). Although all three data sets (native, Pt, and Au) were used for phasing, only the data from the crystal containing Pt revealed heavy-atom positions contributing to phasing power, and the structure was solved essentially by SIRAS methods. Nevertheless, the Au data set was important for getting the best phase estimates and correlation among data sets (including potential anisomorphism between data sets due to the decrease of 0.5 Å in the length of the *c* axis of the soaked crystals), which enabled the phase extension up to 1.80 Å. The quality of the initial electron density map was significantly improved by solvent flattening using SOLOMON (52) through the interface in autoSHARP (53). The results of the data collection and phasing are summarized in Table S4. The solvent-modified phases produced a clear and interpretable electron density map, and the initial model was traced with ARP-WARP (54) with 45 of 134 residues being docked to the electron density. The initial model was improved by visual inspection, and model building was performed with Coot (55). The model was refined to 1.80-Å resolution using CNX (56) and was finally submitted to a last step of TLS (translation, libration, screw) refinement using BUSTER (57) with a final *R*_work_ of 19.3% and *R*_free_ of 21.31 (5% test set) using the parameter set of Engh and Huber (58). The data processing and refinement statistics are summarized in Tables S4 and S5. The coordinates of EVA3 are available from the Protein Data Bank under accession number 6I31.

### Homology modeling

Homology modeling of P1142 mature peptide sequence was performed using MODELLER (25) with PYMOD2.0 plugin in PyMOL (59) using the EVA3 chain A structure as template. Following alignment of P1142 and EVA3 using MUSCLE, homology modeling was performed using default parameters, *i.e.* using all heteroatomic residues, not including water molecules or automatically building or creating disulfides, at default optimization level, and no additional energy minimization, to build 10 models (Fig. S7). The model with the lowest discrete optimized protein energy (DOPE) score was then chosen for further analysis. Solvent-accessible surface and electrostatic potential were modeled using the Adaptive Poisson-Boltzmann Solver (APBS) plugin in PyMOL2.3 (60) using default parameters. Relative surface accessibility was computed as described (61) at the web server ASAView (http://ccbb.jnu.ac.in/shandar/servers/asaview/).^8^

### Statistical analyses

GraphPad Prism was used to calculate summary statistics.

### Ethics approval

All experiments performed on human samples were carried out according to the University of Oxford guidelines following ethics approval (study title, Assays for the development of anti-inflammatory therapeutics; ethics reference, 18/YH/0144).

### Data availability

Data supporting this study are available either here within the text or as supporting information. The coordinates of EVA3 are available from the Protein Data Bank under accession number 6I31. For nucleotide accession numbers, see Table S2.

## Author contributions

A. W. L., M. D., C. L., K. S., J. S., A. E. I. P., J. M. D., and S. B. formal analysis; A. W. L., M. D., C. L., G. D., K. S., Y. A., J. R. O. E., A. K., A. E. I. P., and J. M. D. investigation; A. W. L., M. D., C. L., G. D., K. S., Y. A., J. R. O. E., A. K., J. S., A. E. I. P., and J. M. D. methodology; A. W. L., M. D., C. L., G. D., K. S., Y. A., J. R. O. E., A. K., J. S., A. E. I. P., J. M. D., and S. B. writing-review and editing; M. D., A. E. I. P., and S. B. conceptualization; M. D., A. E. I. P., and S. B. data curation; M. D., J. S., A. E. I. P., J. M. D., and S. B. writing-original draft; G. D., A. K., J. S., A. E. I. P., J. M. D., and S. B. supervision; A. K., A. E. I. P., and S. B. funding acquisition; A. K., A. E. I. P., and S. B. project administration.

## Supplementary Material

Supporting Information

## Footnotes

8 Please note that the JBC is not responsible for the long-term archiving and maintenance of this site or any other third party hosted site.

## Footnotes

7 The abbreviations used are:

CXCR: CXC chemokine receptor
EVA: evasin
BLI: biolayer interferometry
CXCL: C*X*C-motif chemokine ligand
CRS: chemokine recognition site
AF647: Alexa Fluor 647
SIRAS: single isomorphous replacement with anomalous scattering
SUMO: small ubiquitin-like modifier
IMAC: immobilized metal affinity chromatography
APC: allophycocyanin
FSC: forward scatter
SSC: side scatter
EC: effective concentration
Bis-Tris: 2-[bis(2-hydroxyethyl)amino]-2-(hydroxymethyl)propane-1,3-diol.
