# Supplementary material for: A knottin scaffold directs the CXC-chemokine–binding specificity of tick evasins
Source: J Biol Chem. 2019 Jun 5;294(29):11199–212. doi: 10.1074/jbc.RA119.008817 (PMC6643034; doi:10.1074/jbc.RA119.008817)
Supplement: Supporting Information [file supp_294_29_11199__index.html]

A knottin scaffold directs the CXC-chemokine-binding specificity of tick evasins — Knottin scaffold evasins — A knottin scaffold directs the CXC-chemokine–binding specificity of tick evasins — Knottin scaffold evasins — Supporting Information 

# A knottin scaffold directs the CXC-chemokine–binding specificity of tick evasins

## Supporting Information

- Supporting Information (to be published online) - Supporting Information for revised manuscript
