# Supplementary material for: A knottin scaffold directs the CXC-chemokine–binding specificity of tick evasins
Source: J Biol Chem. 2019 Jun 5;294(29):11199–212. doi: 10.1074/jbc.RA119.008817 (PMC6643034; doi:10.1074/jbc.RA119.008817)
Supplement: Supporting Information [file supp_RA119.008817_144833_1_supp_337613_ps28z1.pdf]

Supporting information

A knottin scaffold directs the CXC-chemokine-binding specificity of tick evasins

Angela W. Lee[1]#, Maud Deruaz[2]#, Christopher Lynch[1]#, Graham Davies[1]#, Kamayani Singh[1], Yara Alenazi[1], James R.O. Eaton[1,3], Akane Kawamura[1,3], Jeffrey Shaw[2], Amanda Proudfoot[2], João M. Dias[2*] & Shoumo Bhattacharya[1*].

Contents

Figure S1. Yeast surface display screen using CXCL10.
Figure S2. Expression and purification of CXC-chemokine binding evasins
Figure S3. Cross-binding assays
Figure S4. Summary data of evasin target residence times using biolayer interferometry
Figure S5. Conservation plot of CXC-chemokine binding evasins
Figure S6. KNOTER3D analysis and surface accessibility plots.
Figure S7. Homology modelling statistics
Figure S8. Purification and analysis of segment-swap mutants
Figure S9. Cell migration assays: EC_{80} determination

Table S1. Evasin clones recovered in human CXC chemokine screens
Table S2. Physicochemical properties and predicted glycosylation of evasin proteins
Table S3. Summary of data shown in Fig. S4
Table S4. Data collection and SIRAS Phasing statistics (SHARP) for Evasin-3.
Table S5. Refinement statistics

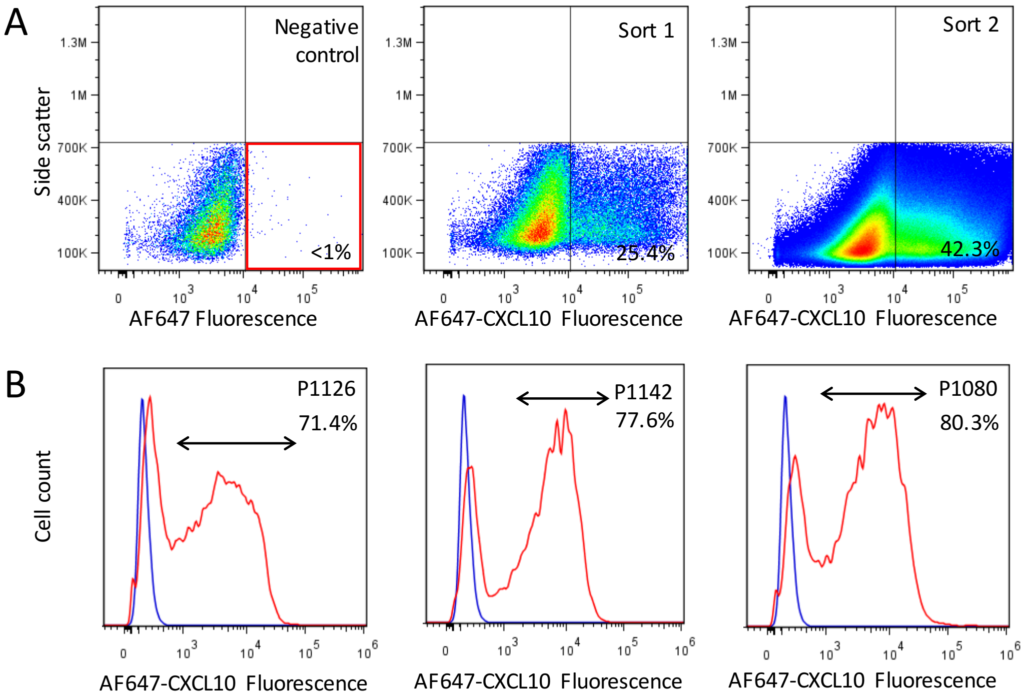

Figure S1: Yeast surface display screen using CXCL10.

A. Fluorescence profiles of yeast surface display library of 6C evasin 3 homologs incubated with streptavidin-AF647 (negative control, most left) and with biotinylated CXCL10 plus streptavidin-AF647 (Sort 1). The sorting gate (red box) was defined based on the negative control and was used to sort evasin-expressing yeast that bound biotinylated CXCL10. The positive cells identified in the first sort were re-grown as a pool, and sorted once again to further enrich for chemokine binding yeast (Sort 2), and plated at low density to recover single clones. The y-axis shows side scatter and x-axis the fluorescence intensity on a log-scale. The proportions of cells within the sorting gate are indicated as a percentage.

B. Representative fluorescence profile (red) confirming the binding of three evasin-expressing yeast clones isolated in the above screen to biotinylated CXCL10 and streptavidin-AF647, including P1126_AMBCA, P1142_AMBCA and P1080_IXORI. The fluorescence profile of control yeast with vector expressing the surface display tag alone are shown in blue. Y-axis shows relative cell count, and x-axis shows the fluorescence intensity on a log-scale. Positive cells with fluorescence exceeding that of the control are indicated as a percentage.

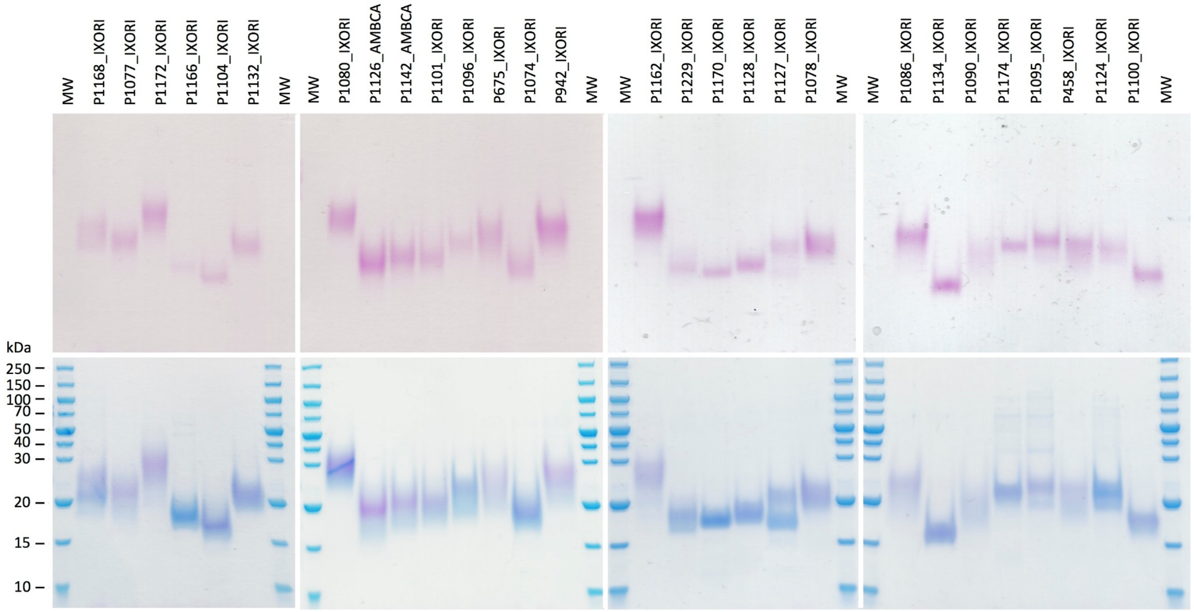

Figure S2. Expression and purification of CXC-chemokine binding evasins

Two µg of each indicated protein was run on a 12% Bis-Tris gel and stained using the enhanced periodic acid-Schiff (PAS) method for detection of glycoprotein sugars (top panels) followed by staining with Coomassie RAPIDstain (lower panels). MW - Molecular weight markers, sizes indicated in kDa.

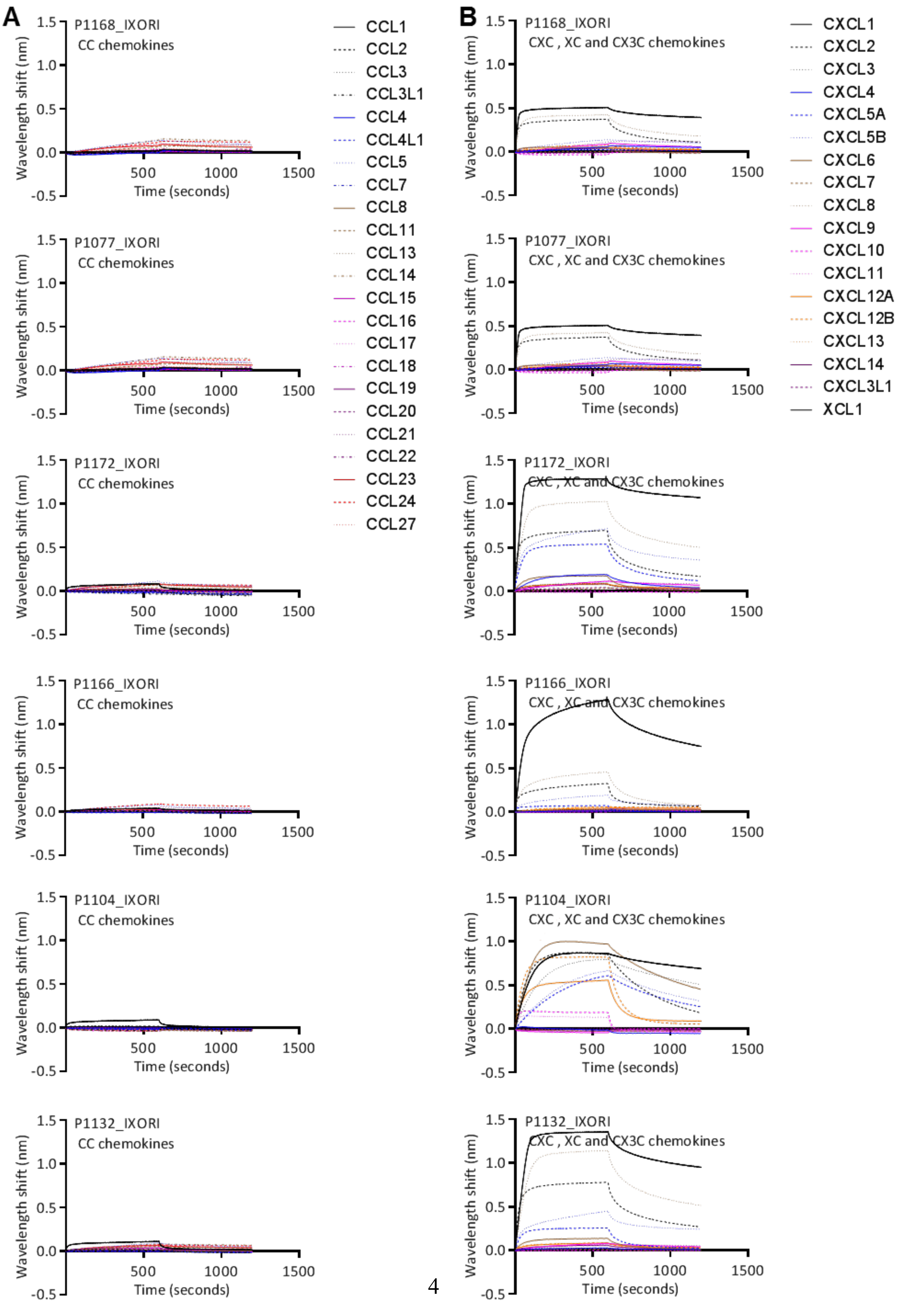
A
B
P1168_IXORI
CC chemokines
P1077_IXORI
P1172_IXORI
P1166_IXORI
P1104_IXORI
P1132_IXORI
CXC , XC and CX3C chemokines
Wavelength shift (nm)
Time (seconds)
CCL1
CCL2
CCL3
CCL3L1
CCL4
CCL4L1
CCL5
CCL7
CCL8
CCL11
CCL13
CCL14
CCL15
CCL16
CCL17
CCL18
CCL19
CCL20
CCL21
CCL22
CCL23
CCL24
CCL27
CXCL1
CXCL2
CXCL3
CXCL4
CXCL5A
CXCL5B
CXCL6
CXCL7
CXCL8
CXCL9
CXCL10
CXCL11
CXCL12A
CXCL12B
CXCL13
CXCL14
CXCL3L1
XCL1

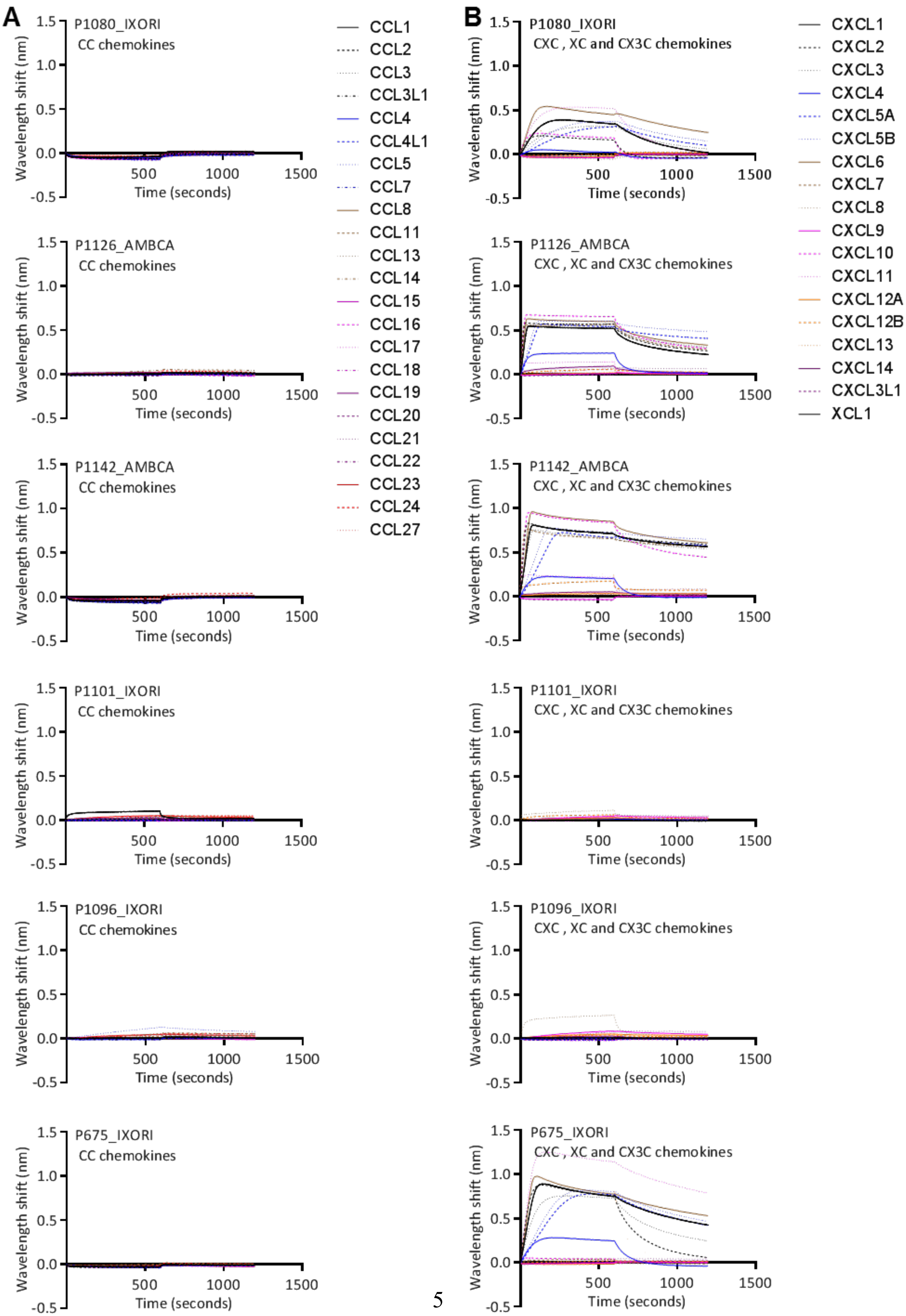
A
B
P1080_IXORI
P1126_AMBCA
P1142_AMBCA
P1101_IXORI
P1096_IXORI
P675_IXORI
CC chemokines
CXC , XC and CX3C chemokines
Wavelength shift (nm)
Time (seconds)
CCL1
CCL2
CCL3
CCL3L1
CCL4
CCL4L1
CCL5
CCL7
CCL8
CCL11
CCL13
CCL14
CCL15
CCL16
CCL17
CCL18
CCL19
CCL20
CCL21
CCL22
CCL23
CCL24
CCL27
CXCL1
CXCL2
CXCL3
CXCL4
CXCL5A
CXCL5B
CXCL6
CXCL7
CXCL8
CXCL9
CXCL10
CXCL11
CXCL12A
CXCL12B
CXCL13
CXCL14
CXCL3L1
XCL1

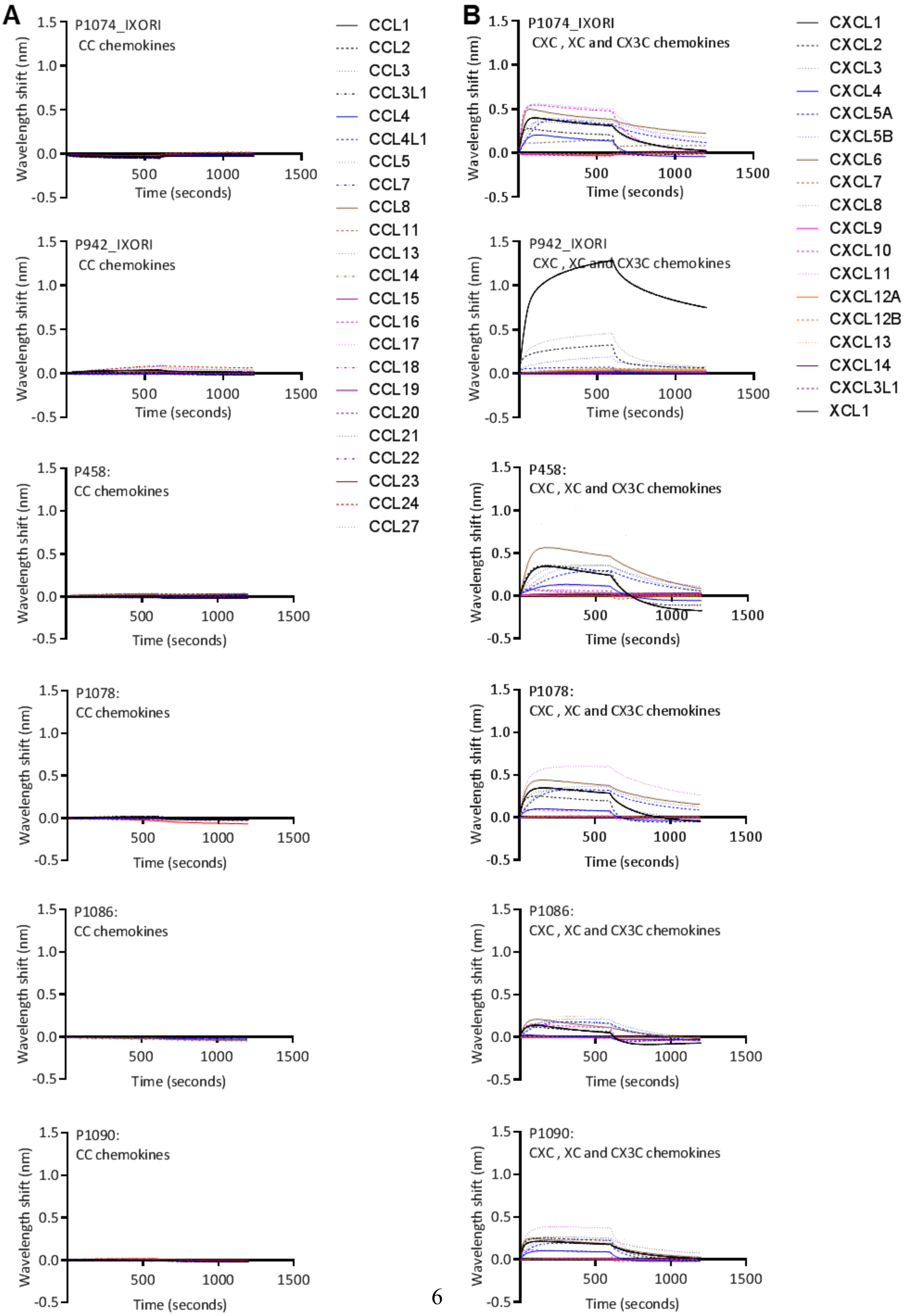

A
P1074_IXORI
CC chemokines
P942_IXORI
CC chemokines
P458:
CC chemokines
P1078:
CC chemokines
P1086:
CC chemokines
P1090:
CC chemokines
Wavelength shift (nm)
Time (seconds)
CCL1
CCL2
CCL3
CCL3L1
CCL4
CCL4L1
CCL5
CCL7
CCL8
CCL11
CCL13
CCL14
CCL15
CCL16
CCL17
CCL18
CCL19
CCL20
CCL21
CCL22
CCL23
CCL24
CCL27
B
P1074_IXORI
CXC , XC and CX3C chemokines
P942_IXORI
CXC , XC and CX3C chemokines
P458:
CXC , XC and CX3C chemokines
P1078:
CXC , XC and CX3C chemokines
P1086:
CXC , XC and CX3C chemokines
P1090:
CXC , XC and CX3C chemokines
CXCL1
CXCL2
CXCL3
CXCL4
CXCL5A
CXCL5B
CXCL6
CXCL7
CXCL8
CXCL9
CXCL10
CXCL11
CXCL12A
CXCL12B
CXCL13
CXCL14
CXCL3L1
XCL1

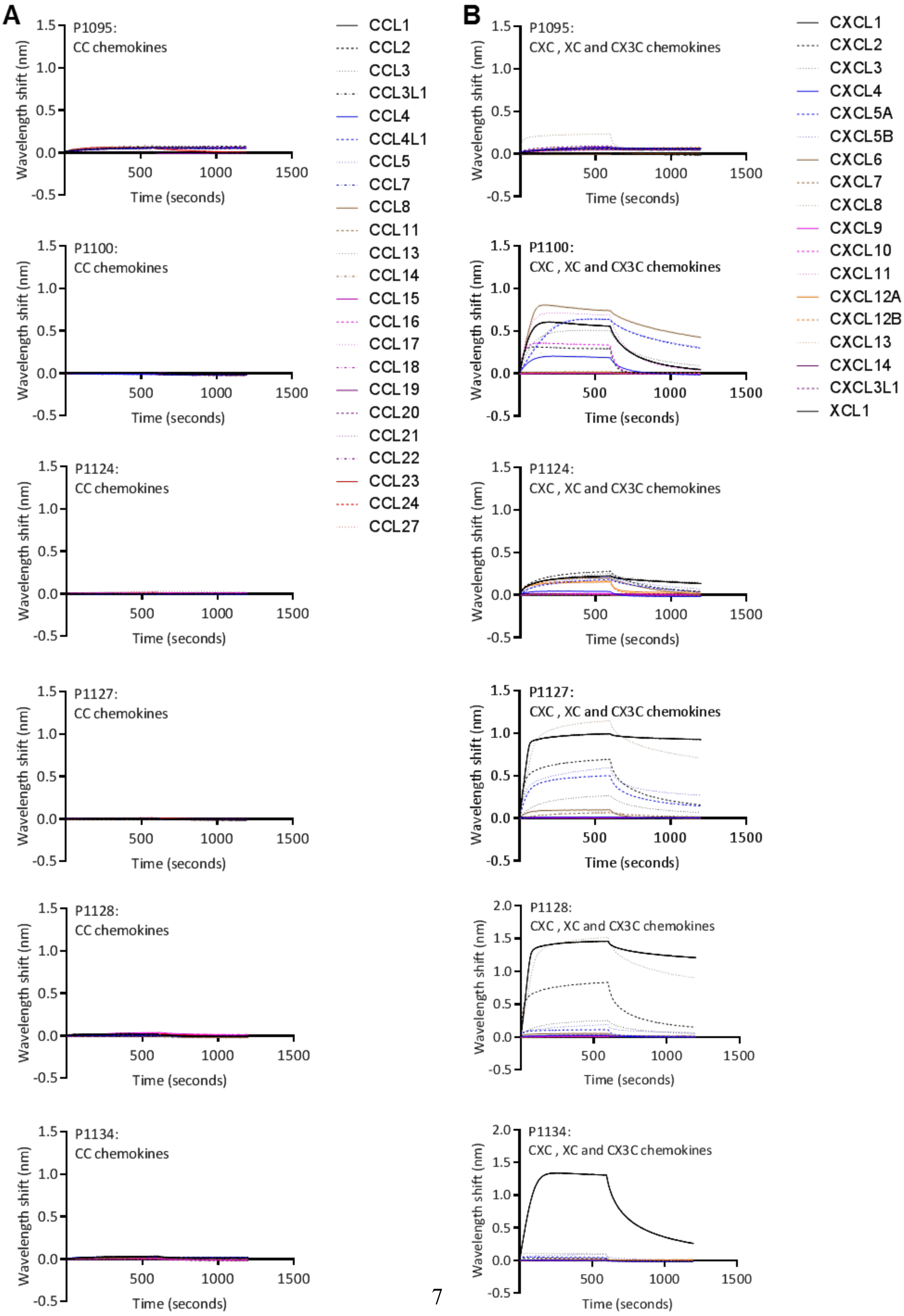

A
B
P1095:
CC chemokines
P1100:
CC chemokines
P1124:
CC chemokines
P1127:
CC chemokines
P1128:
CC chemokines
P1134:
CC chemokines
P1095:
CXC , XC and CX3C chemokines
P1100:
CXC , XC and CX3C chemokines
P1124:
CXC , XC and CX3C chemokines
P1127:
CXC , XC and CX3C chemokines
P1128:
CXC , XC and CX3C chemokines
P1134:
CXC , XC and CX3C chemokines
Wavelength shift (nm)
Time (seconds)
CCL1
CCL2
CCL3
CCL3L1
CCL4
CCL4L1
CCL5
CCL7
CCL8
CCL11
CCL13
CCL14
CCL15
CCL16
CCL17
CCL18
CCL19
CCL20
CCL21
CCL22
CCL23
CCL24
CCL27
CXCL1
CXCL2
CXCL3
CXCL4
CXCL5A
CXCL5B
CXCL6
CXCL7
CXCL8
CXCL9
CXCL10
CXCL11
CXCL12A
CXCL12B
CXCL13
CXCL14
CXCL3L1
XCL1

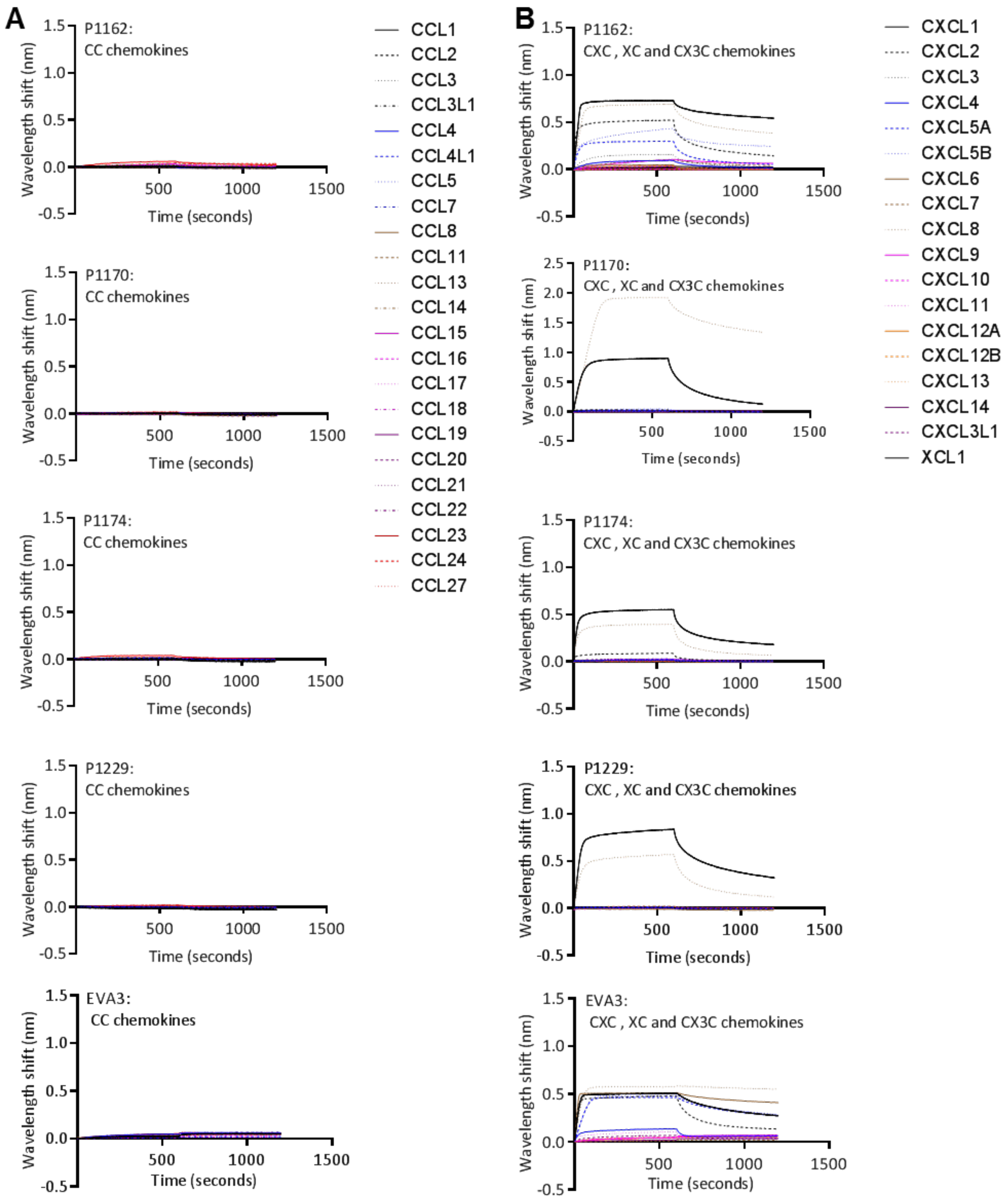

Figure S3. Cross binding assays.

A. Cross binding to CC chemokines for indicated evasins. Biolayer interferometry sensorgrams showing binding to different chemokines. Plots display wavelength shift (y-axis, nm) versus time (x-axis, seconds).

B. Cross binding to CXC, CX3C and XC chemokines for indicated evasins. Biolayer interferometry sensorgrams showing binding to different chemokines at 300 nM. Plots display wavelength shift (y-axis, nm) versus time (x-axis, seconds).

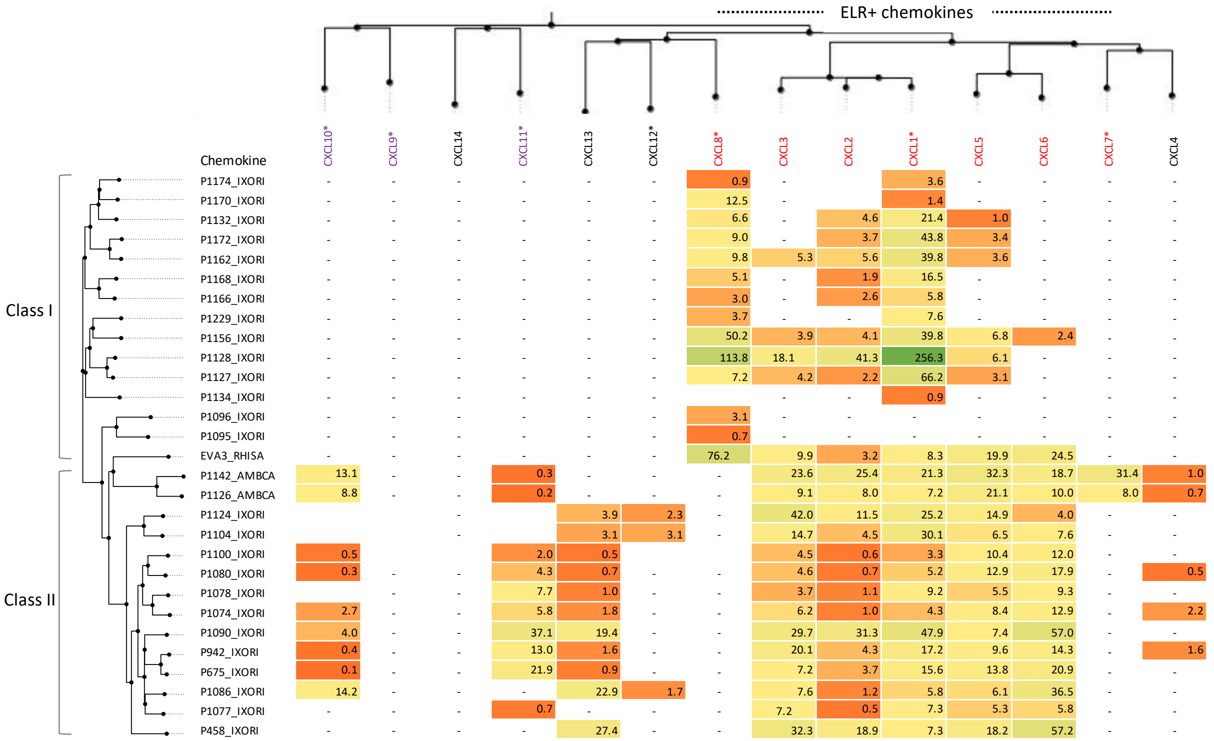

Chemokine	CXCL10*	CXCL9*	CXCL14	CXCL11*	CXCL13	CXCL12*	CXCL8*	CXCL3	CXCL2	CXCL1*	CXCL5	CXCL6	CXCL7*	CXCL4
P1174_IXORI	-	-	-	-	-	-	0.9	-		3.6	-	-	-	-
P1170_IXORI	-	-	-	-	-	-	12.5	-		1.4	-	-	-	-
P1132_IXORI	-	-	-	-	-	-	6.6	-	4.6	21.4	1.0	-	-	-
P1172_IXORI	-	-	-	-	-	-	9.0	-	3.7	43.8	3.4	-	-	-
P1162_IXORI	-	-	-	-	-	-	9.8	5.3	5.6	39.8	3.6	-	-	-
P1168_IXORI	-	-	-	-	-	-	5.1	-	1.9	16.5	-	-	-	-
P1166_IXORI	-	-	-	-	-	-	3.0	-	2.6	5.8	-	-	-	-
P1229_IXORI	-	-	-	-	-	-	3.7	-	-	7.6	-	-	-	-
P1156_IXORI	-	-	-	-	-	-	50.2	3.9	4.1	39.8	6.8	2.4	-	-
P1128_IXORI	-	-	-	-	-	-	113.8	18.1	41.3	256.3	6.1	-	-	-
P1127_IXORI	-	-	-	-	-	-	7.2	4.2	2.2	66.2	3.1	-	-	-
P1134_IXORI	-	-	-	-	-	-		-	-	0.9	-	-	-	-
P1096_IXORI	-	-	-	-	-	-	3.1	-	-	-	-	-	-	-
P1095_IXORI	-	-	-	-	-	-	0.7	-	-	-	-	-	-	-
EVA3_RHISA	-	-	-	-	-	-	76.2	9.9	3.2	8.3	19.9	24.5	-	-
P1142_AMBCA	13.1	-	-	0.3	-	-	-	23.6	25.4	21.3	32.3	18.7	31.4	1.0
P1126_AMBCA	8.8	-	-	0.2	-	-	-	9.1	8.0	7.2	21.1	10.0	8.0	0.7
P1124_IXORI			-		3.9	2.3	-	42.0	11.5	25.2	14.9	4.0	-	-
P1104_IXORI			-		3.1	3.1	-	14.7	4.5	30.1	6.5	7.6	-	-
P1100_IXORI	0.5			2.0	0.5			4.5	0.6	3.3	10.4	12.0	-	-
P1080_IXORI	0.3	-	-	4.3	0.7	-	-	4.6	0.7	5.2	12.9	17.9	-	0.5
P1078_IXORI		-	-	7.7	1.0	-	-	3.7	1.1	9.2	5.5	9.3	-	-
P1074_IXORI	2.7	-	-	5.8	1.8	-	-	6.2	1.0	4.3	8.4	12.9	-	2.2
P1090_IXORI	4.0	-	-	37.1	19.4	-	-	29.7	31.3	47.9	7.4	57.0	-	-
P942_IXORI	0.4	-	-	13.0	1.6	-	-	20.1	4.3	17.2	9.6	14.3	-	1.6
P675_IXORI	0.1	-	-	21.9	0.9	-	-	7.2	3.7	15.6	13.8	20.9	-	-
P1086_IXORI	14.2	-	-	-	22.9	1.7	-	7.6	1.2	5.8	6.1	36.5	-	-
P1077_IXORI	-	-	-	0.7	-	-	-	7.2	0.5	7.3	5.3	5.8	-	-
P458_IXORI	-	-	-	-	27.4	-	-	32.3	18.9	7.3	18.2	57.2	-	-

Figure S4. Summary data of evasin target residence times using biolayer interferometry
Target residence times (min) of immobilized evasin to human CXC-chemokines using biolayer interferometry. Prolonged residence times are indicated as shades of green, medium as yellow, and low as shades of orange. Chemokines and evasins are arranged by sequence-similarity based phylogeny. An asterisk following a chemokine indicates that it was used for yeast surface display screening. ELR+ chemokines are highlighted in red. A dash (-) indicates that binding was not detected at 300 nM chemokine concentration. Data for P1156_IXORI were reported previously(1) and are shown for comparison. Evasin functional classes I and II are indicated, see text for details.

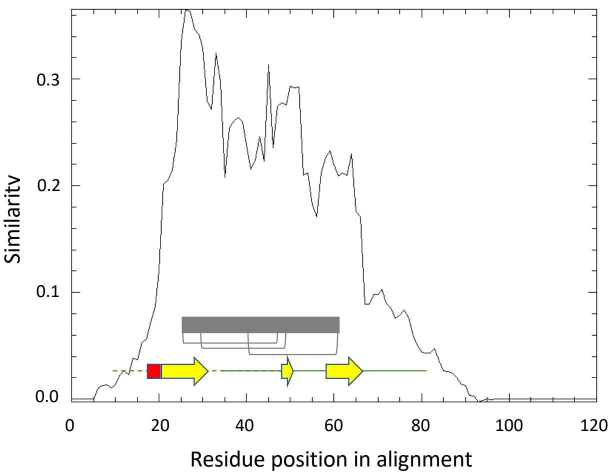

F

Figure S5: Conservation plot of CXC-chemokine binding evasins

Conservation of evasins identified by yeast surface display. The plot was generated from the sequence alignment using the EMBOSS program plotcon with a window size of 10 residues. Y-axis shows similarity score and x-axis shows the residue position in the alignment. The grey bar indicates the disulfide bonded central core. Disulfide bonds (DSB) identified in the evasin 3 X-ray crystal structure are indicated by grey lines. Secondary structures in EVA3 chain A are indicated and are colored as a red bar (α-helix), yellow arrow (β-strands) and green line (loop). The dashed green line indicates residues that are not visible in the EVA3 chain A or chain B X-ray crystal structures.

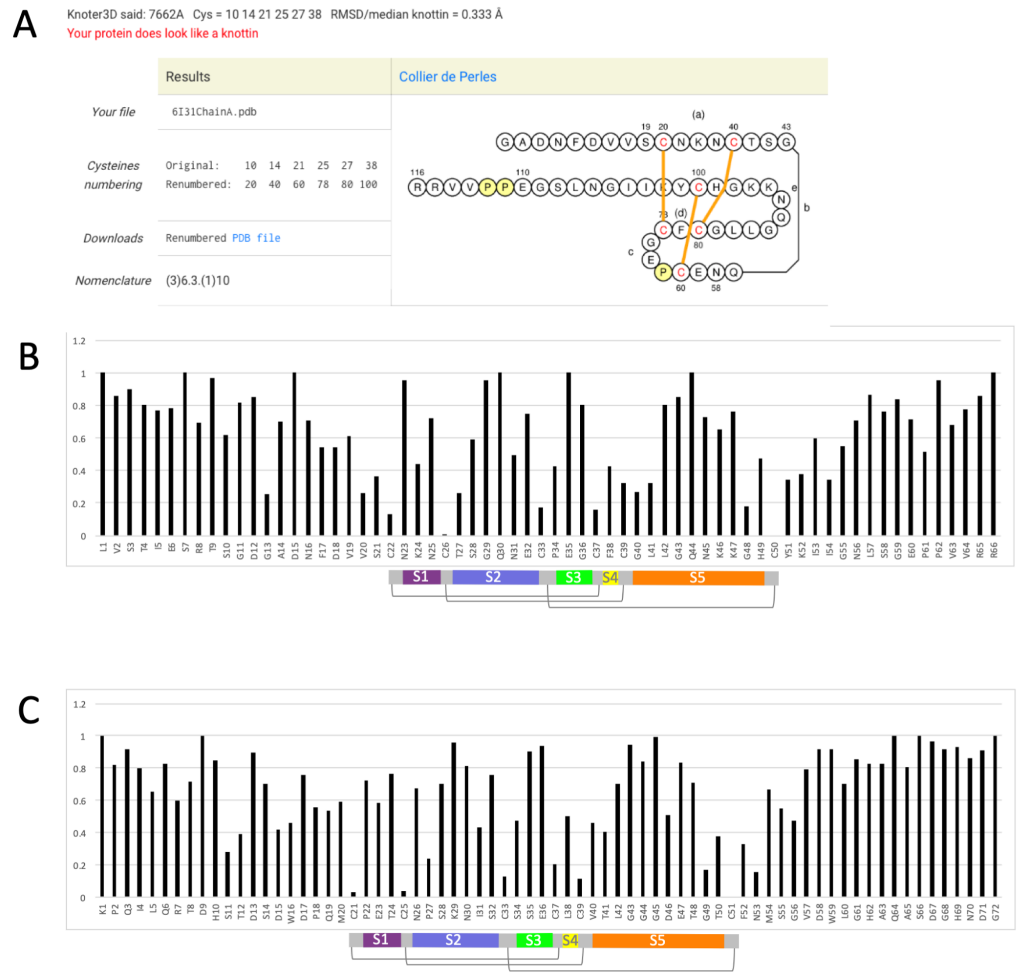

Figure S6. KNOTER3D analysis and surface accessibility plots.

A. KNOTER3D (3) analysis of EVA3 Chain A showing renumbered Cys residues, knottin nomenclature, and a 2D "Collier de Perles" representation of the EVA3 molecule showing disulfide bond connectivity.

B, C. Bar charts showing relative surface accessibility (y-axis) by residue (x-axis) in EVA3 (B) and P1142 (C). Disulfide bonds (DSB) identified in the EVA3 X-ray crystal structure are indicated by grey lines between Cys residues. The grey bar indicates the conserved disulfide bonded central core. Bars indicating segments S1-S5 between Cys residues are colored as violet, blue, green, yellow and orange respectively.

A

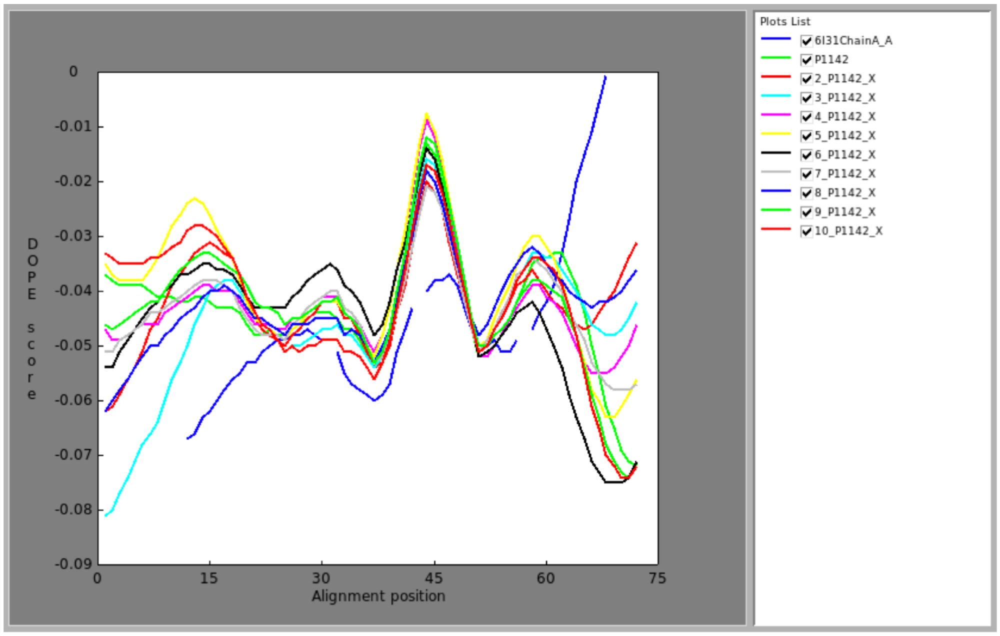

B

	Objective Function Value	DOPE score
P1142.B99990001.pdb	326.651	-3830.695
P1142.B99990002.pdb	368.828	-3787.005
P1142.B99990003.pdb	355.018	-3663.506
P1142.B99990004.pdb	440.15	-3637.765
P1142.B99990005.pdb	343.834	-3565.886
P1142.B99990006.pdb	364.392	-3718.972
P1142.B99990007.pdb	350.151	-3747.525
P1142.B99990008.pdb	355.818	-3704.331
P1142.B99990009.pdb	450.705	-3740.39
P1142.B99990010.pdb	322.184	-3876.025

Figure S7. Homology modelling statistics. **A.** Graph showing DOPE score (y-axis) plotted against the alignment position for 10 models of P1142, compared to Evasin3 Chain A (6I31ChainA) that were generated by MODELLER. **B.** Table of Modeller Objective Function Value and DOPE Score for each P1142 model. Model 10 (P1142_B99999010.pdb) had the lowest DOPE score of -3876, and was used for all further analysis.

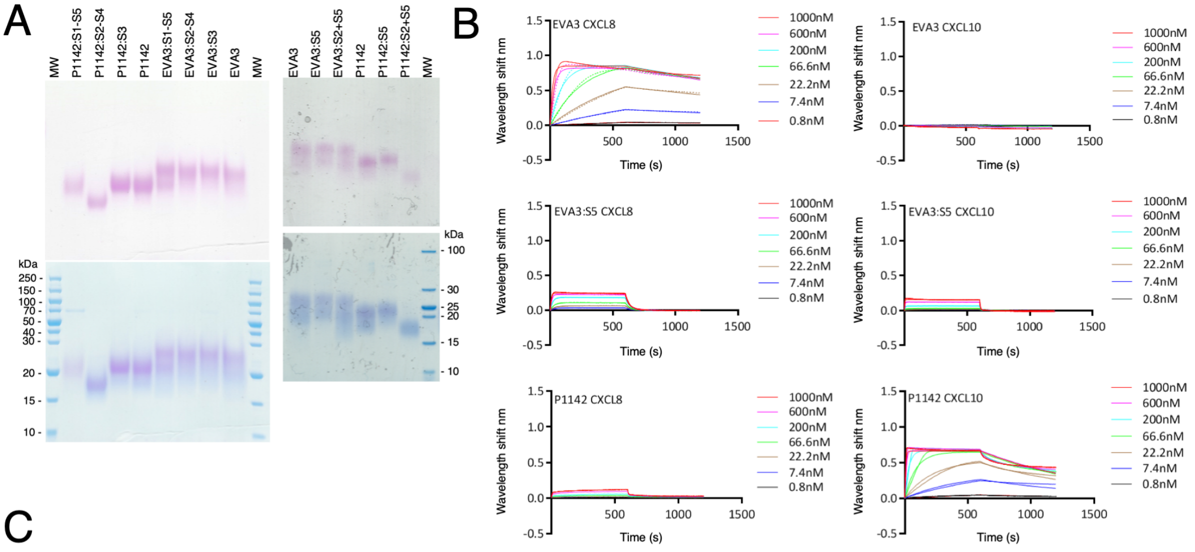

C

	CXCL10	CXCL11	CXCL8	CXCL3	CXCL2	CXCL1	CXCL5	CXCL6	CXCL7	CXCL4
EVA3	-	-	76.24	9.91	3.19	8.27	19.94	24.48	-	-
EVA3:S3	-	-	17.39	4.72	1.21	2.54	5.30	10.02	-	-
EVA3:S2-S4	-	-	0.55	-	-	0.41	4.62	9.19	-	-
EVA3:S1-S5	0.34	-	-	1.97	0.31	0.31	1.58	0.51	2.12	-
EVA3:S5	0.13	0.08	0.56	1.26	0.17	0.73	1.25	0.56	-	-
EVA3:S2+S5	0.14	0.09	0.13	0.55	0.05	0.35	1.13	0.37	0.56	-
P1142:S2+S5	-	-	-	-	-	-	-	-	-	-
P1142:S5	-	-	-	-	-	-	-	-	-	-
P1142:S1-S5	-	-	-	-	-	-	-	-	-	-
P1142:S2-S4	-	0.03	-	8.47	-	-	1.75	0.57	-	-
P1142:S3	3.97	0.08	-	11.31	13.94	11.20	12.09	4.98	14.91	-
P1142	13.15	0.34	-	25.41	21.34	32.32	23.62	18.67	31.43	1.03

Figure S8. Purification and analysis of segment swap mutants

A. Two µg of each indicated protein was run on a 12% Bis-Tris gel and stained using the enhanced periodic acid-Schiff (PAS) method for detection of glycoprotein sugars (top panel) followed by staining with Coomassie RAPIDstain (lower panel). MW - Molecular weight markers, sizes indicated in kDa.

B. Biolayer interferometry sensorgrams showing binding of EVA3, EVA3:S5 and P1142 to different doses of chemokines CXCL8 (left panel) and CXCL10 (right panel). Plots display wavelength shift (Y-axis, nm) versus time (X-axis, seconds). Solid lines indicate collected data, dashed lines indicate fitted data. Note that while the BLI trace suggested weak binding of P1142 to CXCL8 when a high concentration (1000 nM) of chemokine was used, the data could not be fitted.

C. Target residence times (min) of immobilized segment-swap mutants to human CXC-chemokines using biolayer interferometry. Prolonged residence times are indicated as shades of green, medium as yellow, and low as shades of orange. ELR+ chemokines are highlighted in red. A dash (-) indicates that a binding constant could not be calculated, and is interpreted as lack of binding.

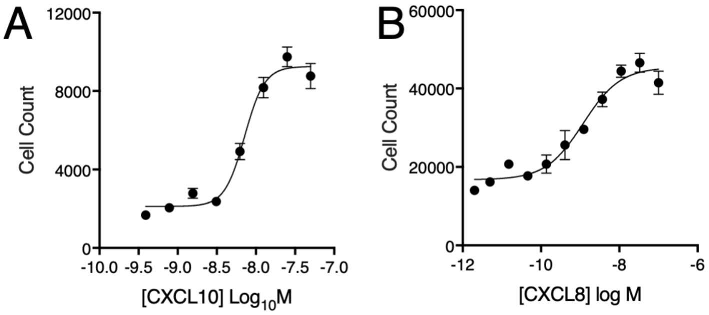

Figure S9. Cell migration assays: EC_{80} determination

A. A representative example of an experiment to determine CXCL10 EC_{80} is shown. The Y-axis shows human activated T-cell count migrating through to the bottom chamber in response to increasing doses of human CXCL10. Data (mean ± s.e.m of three technical replicates) is shown. X-axis shows CXCL10 concentration (Log_{10} Molar). EC_{80} (1.08E-8M) was estimated by fitting an agonist response curve with 4 parameters as described(4).

B. A representative example of an experiment to determine CXCL8 EC_{80} is shown. The Y-axis shows the count of human granulocytes extracted from buffy coat migrating through to the bottom chamber in response to increasing doses of human CXCL8. Data (mean ± s.e.m of three technical replicates) is shown. X-axis shows CXCL8 concentration (Log_{10} Molar). EC_{80} (5.42E-9M) was estimated by fitting an agonist response curve with 4 parameters as described(4).

Table S1. Evasin clones recovered in human CXC chemokine screens. ELR+ chemokines used in the screen are indicated in red font (column 1). Evasin protein sequence (column 2) prefix indicates the identity, and suffix indicate the tick species as follows: RHISA - *Rhipicephalus sanguineus*, AMBCA, *Amblyomma cajennense,* IXORI - *Ixodes ricinus*. The number of yeast clones for each evasin recovered in a yeast surface display screen (using a particular chemokine) and then re-tested is shown in column 3. The mean percentage of yeast cells exceeding control threshold fluorescence (yeast labelled with streptavidin-AF647 alone), averaged over the clones re-tested, is indicated in column 4. The outcome of validation by BLI (biolayer interferometry) for each interaction detected by yeast surface display is indicated in columns 5 and 6.

Chemokine	Evasin identified	Number of clones recovered and re-tested	Mean % cells over control threshold fluorescence	Validation by BLI for binding to screen chemokine	Validation by BLI for binding to any chemokine
CXCL1_HUMAN	EVA3_RHISA	2	69.15	Yes	Yes
CXCL1_HUMAN	P1077_IXORI	6	49.70	Yes	Yes
CXCL1_HUMAN	P1104_IXORI	2	64.63	Yes	Yes
CXCL1_HUMAN	P1132_IXORI	1	68.32	Yes	Yes
CXCL1_HUMAN	P1156_IXORI	3	58.79	Yes	Yes
CXCL1_HUMAN	P1162_IXORI	1	71.69	Yes	Yes
CXCL1_HUMAN	P1166_IXORI	2	63.30	Yes	Yes
CXCL1_HUMAN	P1168_IXORI	9	65.58	Yes	Yes
CXCL1_HUMAN	P1172_IXORI	2	64.89	Yes	Yes
CXCL1_HUMAN	P1229_IXORI	1	66.17	Yes	Yes
CXCL7_HUMAN	P1126_AMBCA	8	73.74	Yes	Yes
CXCL7_HUMAN	P1142_AMBCA	8	71.38	Yes	Yes
CXCL8_HUMAN	EVA3_RHISA	9	80.99	Yes	Yes
CXCL8_HUMAN	P1127_IXORI	1	42.53	Yes	Yes
CXCL8_HUMAN	P1128_IXORI	1	53.20	Yes	Yes
CXCL8_HUMAN	P1156_IXORI	9	67.01	Yes	Yes
CXCL8_HUMAN	P1168_IXORI	1	23.76	Yes	Yes
CXCL8_HUMAN	P1170_IXORI	1	80.91	Yes	Yes
CXCL8_HUMAN	P1172_IXORI	1	41.47	Yes	Yes
CXCL8_HUMAN	P1174_IXORI	1	29.34	Yes	Yes
CXCL9_HUMAN	P458_IXORI	2	37.28	No	Yes
CXCL9_HUMAN	P1074_IXORI	1	34.97	No	Yes
CXCL9_HUMAN	P1080_IXORI	2	49.35	No	Yes
CXCL9_HUMAN	P1086_IXORI	1	54.09	No	Yes
CXCL9_HUMAN	P1100_IXORI	2	55.44	No	Yes
CXCL9_HUMAN	P1124_IXORI	1	31.12	No	Yes
CXCL9_HUMAN	P1126_AMBCA	5	55.63	No	Yes
CXCL10_HUMAN	P1074_IXORI	2	70.97	Yes	Yes
CXCL10_HUMAN	P1080_IXORI	11	69.72	Yes	Yes
CXCL10_HUMAN	P1086_IXORI	2	63.03	Yes	Yes
CXCL10_HUMAN	P1096_IXORI	1	50.27	No	Yes
CXCL10_HUMAN	P1101_IXORI	1	74.97	No	No
CXCL10_HUMAN	P1126_AMBCA	3	65.93	Yes	Yes
CXCL10_HUMAN	P1134_IXORI	2	65.87	No	Yes
CXCL10_HUMAN	P1142_AMBCA	1	79.65	Yes	Yes
CXCL11_HUMAN	P675_IXORI	1	54.60	Yes	Yes
CXCL11_HUMAN	P942_IXORI	2	37.12	Yes	Yes
CXCL11_HUMAN	P1074_IXORI	2	33.15	Yes	Yes
CXCL11_HUMAN	P1078_IXORI	1	38.17	No	Yes
CXCL11_HUMAN	P1080_IXORI	5	45.33	Yes	Yes
CXCL11_HUMAN	P1090_IXORI	3	31.85	Yes	Yes
CXCL12_HUMAN	P1080_IXORI	18	53.17	No	Yes
CXCL12_HUMAN	P1095_IXORI	1	47.17	No	Yes

Table S2. Physicochemical properties and predicted glycosylation of evasin proteins
Abbreviations: MW – molecular weight, Da. Percentage identity (to EVA3, EVA4 or EVA1) over alignment length in residues were calculated using BLAST. Length refers to number of amino acid residues. Predicted glycosylation sites were identified as described in methods.

Evasin	Accession	% Identity to EVA3, EVA4 or EVA1 over alignment length in residues	pI	MW Da	Length (amino acid residues)	N-glycosylated residue position	O-glycosylated residue position
P458_IXORI	JAA66389.1	EVA3_RHISA: 33%_57	4.16	6715	66	23	2,12
P675_IXORI	JAA65288.1	EVA3_RHISA: 34%_53	3.85	7026	68	23	2,6,11,14
P942_IXORI	JAA66316.1	EVA3_RHISA: 32%_53	3.69	6988	66	21	4,9,12
P1074_IXORI	JAA69185.1	EVA3_RHISA: 27%_56	4.76	7496	68	46	2,5,6
P1077_IXORI	JAC92493.1	EVA3_RHISA: 42%_38	4.44	7188	68	46	2,11,12,14,56
P1078_IXORI	JAA66284.1	EVA3_RHISA: 32%_47	6.04	7359	68	23,46	2,5,6,10,11
P1080_IXORI	JAC92495.1	EVA3_RHISA: 33%_40	8.51	11025	99	48,85	1,4,7,8,66,68,69, 75,87,88
P1086_IXORI	JAA71238.1	EVA3_RHISA: 30%_40	4.22	7134	66		
P1090_IXORI	JAC92450.1	EVA3_RHISA: 31%_45	4.12	6127	61	46	11
P1095_IXORI	JAA68746.1	EVA3_RHISA: 37%_49	4.47	9751	89	28	2,7,13,78,81
P1096_IXORI	JAA71014.1		4.40	9769	91	27	2,3,5,7,81,83,87
P1100_IXORI	JAA66297.1	EVA3_RHISA: 36%_45	4.87	7213	68	23,46	2,5,6,8,14
P1104_IXORI	JAC91915.1	EVA3_RHISA: 29%_63	4.37	6863	66	23	2,6,8
P1124_IXORI	JAA69499.1	EVA3_RHISA: 30%_47	4.16	5749	55	23	
P1126_AMBCA	JAC19589.1	EVA3_RHISA: 31%_52	4.05	7127	65	52	
P1127_IXORI	JAA71111.1	EVA3_RHISA: 30%_50	4.76	8768	80	16,61	71
P1128_IXORI	JAA66418.1	EVA3_RHISA: 29%_45	4.54	7978	72	14,59	69
P1132_IXORI	JAB69609.1		4.84	8735	78	16,31	60,61
P1134_IXORI	JAA68125.1	EVA3_RHISA: 34%_35	6.90	11961	104	13	58,60,62,68,74
P1142_AMBCA	JAC18880.1		4.26	7687	72	26,53	
P1162_IXORI	JAA69121.1	EVA4_RHISA: 37%_35; EVA3_RHISA: 34%_38	3.79	7928	71	15,21,30,57	
P1166_IXORI	JAA68804.1		5.09	9560	86	24	4,73
P1168_IXORI	JAA68901.1	EVA3_RHISA: 27%_44	4.32	9099	82	24,30,40	4
P1170_IXORI	JAA69006.1	EVA3_RHISA: 33%_49	4.64	8468	74	16	
P1172_IXORI	JAB83717.1	EVA3_RHISA: 34%_38; EVA4_RHISA: 34%_35	4.08	7941	71	15,21,30,57	
P1174_IXORI	JAB82333.1	EVA4_RHISA: 26%_31	4.8	8704	77	16,22,31	60,61,68
P1229_IXORI	JAA66056.1		4.17	8466	77	13	62,68,75

Table S3. Summary of data shown in Fig. S4

Evasin	Chemokines bound at 300 nM
**P1168_IXORI**	**CC, XC, and CX3C:** None<br>**CXC:** CXCL1, CXCL2, CXCL8
**P1077_IXORI**	**CC, XC, and CX3C:** None<br>**CXC**: CXCL1, CXCL2, CXCL3, CXCL5, CXCL6, CXCL11
**P1172_IXORI**	**CC, XC, and CX3C:** None<br>**CXC:** CXCL1, CXCL2, CXCL5, CXCL8
**P1166_IXORI**	**CC, XC, and CX3C:** None<br>**CXC**: CXCL1, CXCL2, CXCL8
**P1104_IXORI**	**CC, XC, and CX3C:** None<br>**CXC:** CXCL1, CXCL2, CXCL3, CXCL5, CXCL6, CXCL12, CXCL13
**P1132_IXORI**	**CC, XC, and CX3C:** None<br>**CXC**: CXCL1, CXCL2, CXCL5, CXCL8
**P1080_IXORI**	**CC, XC, and CX3C:** None<br>**CXC:** CXCL1, CXCL2, CXCL3, CXCL4, CXCL5, CXCL6, CXCL10, CXCL11, CXCL13
**P1126_AMBCA**	**CC, XC, and CX3C:** None<br>**CXC:** CXCL1, CXCL2, CXCL3, CXCL4, CXCL5, CXCL6, CXCL7, CXCL10, CXCL11
**P1142_AMBCA**	**CC, XC, and CX3C:** None<br>**CXC:** CXCL1, CXCL2, CXCL3, CXCL4, CXCL5, CXCL6, CXCL7, CXCL10, CXCL11
**P1101_IXORI**	**CC, XC, and CX3C:** None<br>**CXC:** None
**P1096_IXORI**	**CC, XC, and CX3C:** None<br>**CXC:** CXCL8
**P675_IXORI**	**CC, XC, and CX3C:** None

	**CXC:** CXCL1, CXCL2, CXCL3, CXCL4, CXCL5, CXCL6, CXCL10, CXCL11, CXCL13
**P1074_IXORI**	**CC, XC, and CX3C:** None<br>**CXC:** CXCL1, CXCL8
**P942_IXORI**	**CC, XC, and CX3C:** None<br>**CXC:** CXCL1, CXCL2, CXCL3, CXCL4, CXCL5, CXCL6, CXCL10, CXCL11, CXCL13
**P458_IXORI**	**CC, XC, and CX3C:** None<br>**CXC:** CXCL1, CXCL2, CXCL3, CXCL5, CXCL6, CXCL13
**P1078_IXORI**	**CC, XC, and CX3C:** None<br>**CXC:** CXCL1, CXCL2, CXCL3, CXCL5, CXCL6, CXCL11, CXCL13
**P1086_IXORI**	**CC:** None<br>**CXC:** CXCL1, CXCL2, CXCL3, CXCL5, CXCL6, CXCL10, CXCL12, CXCL13
**P1090_IXORI**	**CC, XC, and CX3C:** None<br>**CXC:** CXCL1, CXCL2, CXCL3, CXCL5, CXCL6, CXCL10, CXCL11, CXCL13
**P1095_IXORI**	**CC, XC, and CX3C:** None<br>**CXC:** CXCL8
**P1100_IXORI**	**CC, XC, and CX3C:** None<br>**CXC:** CXCL1, CXCL2, CXCL3, CXCL5, CXCL6, CXCL10, CXCL11, CXCL13
**P1124_IXORI**	**CC, XC, and CX3C:** None<br>**CXC:** CXCL1, CXCL2, CXCL3, CXCL5, CXCL6, CXCL12, CXCL13
**P1127_IXORI**	**CC, XC, and CX3C:** None<br>**CXC:** CXCL1, CXCL2, CXCL3, CXCL5, CXCL8
**P1128_IXORI**	**CC, XC, and CX3C:** None<br>**CXC:** CXCL1, CXCL2, CXCL3, CXCL5, CXCL8
**P1134_IXORI**	**CC, XC, and CX3C:** None<br>**CXC:** CXCL1

**P1162_IXORI**	**CC, XC, and CX3C:** None<br>**CXC:** CXCL1, CXCL2, CXCL3, CXCL5, CXCL8
**P1170_IXORI**	**CC, XC, and CX3C:** None<br>**CXC:** CXCL1, CXCL8
**P1174_IXORI**	**CC, XC, and CX3C:** None<br>**CXC:** CXCL1, CXCL8
**P1229_IXORI**	**CC, XC, and CX3C:** None<br>**CXC:** CXCL1, CXCL8
**EVA3_RHISA**	**CC, XC, and CX3C:** None<br>**CXC:** CXCL1, CXCL2, CXCL3, CXCL5, CXCL6, CXCL8

Table S4 Data collection and SIRAS Phasing statistics (SHARP) for EVA3.

Data set	Native	K_2PtCl_4	$KAuCl_4$
Space group	$P3_121$		
Cell parameters	55.09, 55.09, 71.04	54.56, 54.56, 71.85	54.44, 54.44, 71.71
Wavelength (Å)	0.977	0.977	0.977
Resolution (Å)	47.62-1.79	47.14-1.96	47.09-1.79
Total observations	69591	61721	126822
Unique reflections	12035	8852	11894
I/σ	15.0 (2.5)	17.6 (4.4)	19.7 (5.4)
Rsym (%)	6.6 (44.0)	6.9 (32.8)	9.3 (56.6)
Completeness (%)	98.8 (92.9)	96.0 (74.3)	99.4 (97.8)
Redundancy	5.8	7.0	10.7
Heavy atom sites		4 Pt	0
R_{cullis} centric/acentric		0.07/0.08	0.29/0.28
Anomalous R_{cullis}		0.96	0.85
Phasing power centric/acentric		3.00/2.55	0/0
Anomalous Phasing Power		0.37	0
FOM SHARP	0.15		
FOM after solvent flattening and phase extension to 1.80 Å	0.81		

Table S5. Refinement statistics*

Resolution (Å)	15.0 – 1.79
Reflections	11933
Molecules /ASU	2
No. Protein residues/atoms/solvent	99 / 729 / 97
R_{work} overall (1.97Å-1.79Å) (%)	19.1 (19.35)
R_{free} overall (1.97Å-1.79Å) (%)	21.3 (23.13)
RMSD bond lengths (Å) / angles (°)	0.01 / 1.13
Cross-validated Luzzati error (Å)	0.24
Ramachandran plot<br><br>Most favored/additional (%)<br>Generous/disallowed (%)	 <br>95.79 / 4.21<br>0 / 0
Evasin-3 B-values ($Å^2$)	42.33

*Values as defined in BUSTER (5)

1. Alenazi, Y., Singh, K., Davies, G., Eaton, J. R. O., Elders, P., Kawamura, A., and Bhattacharya, S. (2018) Genetically engineered two-warhead evasins provide a method to achieve precision targeting of disease-relevant chemokine subsets. *Sci Rep* **8**, 6333
2. Zlotnik, A., and Yoshie, O. (2012) The chemokine superfamily revisited. *Immunity* **36**, 705-716
3. Postic, G., Gracy, J., Perin, C., Chiche, L., and Gelly, J. C. (2018) KNOTTIN: the database of inhibitor cystine knot scaffold after 10 years, toward a systematic structure modeling. *Nucleic Acids Res* **46**, D454-D458
4. Singh, K., Davies, G., Alenazi, Y., Eaton, J. R. O., Kawamura, A., and Bhattacharya, S. (2017) Yeast surface display identifies a family of evasins from ticks with novel polyvalent CC chemokine-binding activities. *Sci Rep* **7**, 4267
5. Smart, O. S., Womack, T. O., Flensburg, C., Keller, P., Paciorek, W., Sharff, A., Vonrhein, C., and Bricogne, G. (2012) Exploiting structure similarity in refinement: automated NCS and target-structure restraints in BUSTER. *Acta Crystallogr D Biol Crystallogr* **68**, 368-380
